# Evaluation of the Cost-effectiveness of Services for Schizophrenia in the UK Across the Entire Care Pathway in a Single Whole-Disease Model

**DOI:** 10.1001/jamanetworkopen.2020.5888

**Published:** 2020-05-27

**Authors:** Huajie Jin, Paul Tappenden, James H. MacCabe, Stewart Robinson, Sarah Byford

**Affiliations:** 1 King’s Health Economics, Institute of Psychiatry, Psychology, and Neuroscience, King’s College London, London, United Kingdom; 2 Health Economics and Decision Science, University of Sheffield School of Health and Related Research, Sheffield, United Kingdom; 3 Department of Psychosis Studies, Institute of Psychiatry, Psychology, and Neuroscience, King’s College London, London, United Kingdom; 4 Loughborough University School of Business and Economics, Loughborough, United Kingdom

## Abstract

**Importance:**

The existing economic models for schizophrenia often have 3 limitations; namely, they do not cover nonpharmacologic interventions, they report inconsistent conclusions for antipsychotics, and they have poor methodologic quality.

**Objectives:**

To develop a whole-disease model for schizophrenia and use it to inform resource allocation decisions across the entire care pathway for schizophrenia in the UK.

**Design, Setting, and Participants:**

This decision analytical model used a whole-disease model to simulate the entire disease and treatment pathway among a simulated cohort of 200 000 individuals at clinical high risk of psychoses or with a diagnosis of psychosis or schizophrenia being treated in primary, secondary, and tertiary care in the UK. Data were collected March 2016 to December 2018 and analyzed December 2018 to April 2019.

**Exposures:**

The whole-disease model used discrete event simulation; its structure and input data were informed by published literature and expert opinion. Analyses were conducted from the perspective of the National Health Service and Personal Social Services over a lifetime horizon. Key interventions assessed included cognitive behavioral therapy, antipsychotic medication, family intervention, inpatient care, and crisis resolution and home treatment team.

**Main Outcomes and Measures:**

Life-time costs and quality-adjusted life-years.

**Results:**

In the simulated cohort of 200 000 individuals (mean [SD] age, 23.5 [5.1] years; 120 800 [60.4%] men), 66 400 (33.2%) were not at risk of psychosis, 69 800 (34.9%) were at clinical high risk of psychosis, and 63 800 (31.9%) had psychosis. The results of the whole-disease model suggest the following interventions are likely to be cost-effective at a willingness-to-pay threshold of £20 000 ($25 552) per quality-adjusted life-year: practice as usual plus cognitive behavioral therapy for individuals at clinical high risk of psychosis (probability vs practice as usual alone, 0.96); a mix of hospital admission and crisis resolution and home treatment team for individuals with acute psychosis (probability vs hospital admission alone, 0.99); amisulpride (probability vs all other antipsychotics, 0.39), risperidone (probability vs all other antipsychotics, 0.30), or olanzapine (probability vs all other antipsychotics, 0.17) combined with family intervention for individuals with first-episode psychosis (probability vs family intervention or medication alone, 0.58); and clozapine for individuals with treatment-resistant schizophrenia (probability vs other medications, 0.81).

**Conclusions and Relevance:**

The results of this study suggest that the current schizophrenia service configuration is not optimal. Cost savings and/or additional quality-adjusted life-years may be gained by replacing current interventions with more cost-effective interventions.

## Key Points

**Question:**

Which interventions are cost-effective for the prevention and treatment of schizophrenia?

**Findings:**

In this decision analytical model using a simulated cohort of 200 000 individuals, the following interventions were found to be cost-effective: practice as usual plus cognitive behavioral therapy for individuals at clinical high risk of psychosis; a mix of hospital admission and crisis resolution and home treatment team for individuals with acute psychosis; receipt of amisulpride, risperidone, or olanzapine combined with family intervention for individuals with first-episode psychosis; and receipt of clozapine for individuals with treatment-resistant schizophrenia.

**Meaning:**

The results of this study suggest that cost savings and/or additional quality-adjusted life years may be gained by replacing current interventions with more cost-effective interventions.

## Teaser

This decision analytical model develops a whole-disease model for schizophrenia and uses it to inform resource allocation decisions across the entire care pathway for schizophrenia in the UK.

## Introduction

Economic models have increasingly been used to inform decision-making regarding health care, as they provide an explicit way of synthesizing all available data to simulate the likely costs and consequences of using alternative interventions under scenarios that cannot be directly observed in the real world.^1^ A 2020 systematic review found several limitations of existing economic models for schizophrenia.^2^ Most existing models (83%) focused on antipsychotic medications, while there was a lack of models for nonpharmacologic interventions, such as cognitive behavioral therapy (CBT), family intervention, and crisis resolution and home treatment team (CRHT). Second, no antipsychotic medication was shown to be clearly cost-effective compared with the others because of inconsistent or even contradictory conclusions reported by different studies. Third, the quality of existing models was considered low. This systematic review highlighted issues relating to inconsistent assumptions and uses of evidence, which negatively affect the quality of existing economic studies in schizophrenia. Greater consistency could be achieved through the development of generic models that have been agreed on by key stakeholders. A whole-disease model (WDM) represents a type of generic model that is unique in that it can be used to inform multiple resource allocation decisions across the entire care pathway.

Whole-disease models are large-scale models that involve simulating whole disease and treatment pathways, thereby allowing for the economic evaluation of options for the prevention, early identification, diagnosis, treatment, and follow-up of a given disease using a single consistent model.^3^ This type of system-level modeling approach has been successfully applied to a number of disease areas, including cancer, metabolic diseases, and cardiovascular diseases.^4^ However, such an approach has not been applied to any mental health disorders.^4^ The aim of this study was to develop a WDM for schizophrenia services and use it to assess the cost-effectiveness of a range of interventions in the UK.

## Methods

This study was reported according to the Consolidated Health Economic Evaluation Reporting Standards (CHEERS) reporting guideline for reporting health economic evaluations.^5^ Per the Common Rule, ethical approval and informed patient consent were not required given that this is a modeling study with no direct patient contact or influence on patient care directly related to this work.

The methods for developing the schizophrenia WDM were mainly informed by the methodologic framework set out by Tappenden et al.^3^ To help with the development and validation of the WDM, a group of 13 multidisciplinary stakeholders was convened though snowball sampling. The background of the 13 stakeholders included health care professionals practicing in the National Health Service (9 [69.2%]), academic researchers with expertise in mental health economic evaluation (12 [92.3%]), commissioners of mental health services (5 [38.5%]), and service users (2 [15.4%]).

### Population

The target population for the model was individuals referred to secondary care mental health services in the UK because of psychotic symptoms, with a mean (SD) age of 23.5 (5.1) years and a sex ratio of 1.5:1 (men to women).^6^ Of those referred, 33.2% were not at risk of psychosis, 34.9% were at clinical high risk of psychosis (CHR-P), and 31.9% were individuals with psychosis.^6^ Those not at risk of psychosis were included because they also use resources associated with schizophrenia services (eg, specialist assessments).

### Decision Problems

The decision problems to be addressed by the WDM were identified from the scope of the schizophrenia clinical guidelines developed by the National Institute for Health and Care Excellence (NICE).^7,8^ A total of 5 topics were identified (Table 1), which span most of the breadth of the schizophrenia pathway, ranging from the use of CBT for individuals at CHR-P to antipsychotic medication for individuals with treatment-resistant schizophrenia (TRS).

**Table 1.** Decision Problems Addressed Using the Schizophrenia Whole-Disease Model

Topic	Interventions and comparators
Interventions for patients at CHR-P	Practice as usual
	Practice as usual plus 16 sessions of CBT
Interventions for individuals with acute psychosis	Hospital admission alone
	Mix of hospital admission and CRHT
First-line oral antipsychotic medication for FEP	Amisulpride
	Aripiprazole
	Haloperidol
	Olanzapine
	Placebo
	Quetiapine
	Risperidone
Family intervention for FEP	Antipsychotic medication alone
	Family intervention alone
	Antipsychotic medication plus 20 sessions of family intervention
First-line oral antipsychotic medication for TRS	Clozapine
	Haloperidol
	Olanzapine
	Quetiapine
	Risperidone

Abbreviations: CHR-P, clinical high risk of psychosis; CRHT, crisis resolution and home treatment team; CBT, cognitive behavioral therapy; FEP, first-episode psychosis; TRS, treatment-resistant schizophrenia.

### Outcomes and Cost Perspective

In accordance with the NICE Reference Case for economic evaluations,^1^ outcomes were valued in terms of quality-adjusted life-years (QALYs), which are the product of health-related quality of life and quantity of life lived (ie, survival). Costs included those relevant to the National Health Service and Personal Social Services. Costs were reported in 2016 to 2017 UK pounds. Both costs and QALYs were discounted at an annual rate of 3.5%.^1^

### Statistical Analysis

#### Model Design and Implementation

For each intervention under assessment, the consequences of treatment were grouped into the following 4 categories: clinical benefits (eg, preventing relapse), clinical harms (eg, adverse effects), costs (eg, cost of providing the intervention and treating its adverse effects), and cost savings (eg, reduced cost of treating relapse). Not all consequences of interventions were included; common reasons for exclusion were that the treatment was not expected to affect patient outcomes and there was a lack of evidence. For example, clinical harms were only modeled for antipsychotic medications, not for psychosocial interventions because of a lack of adverse event data for nonpharmacologic interventions. The key consequences of all interventions included in the schizophrenia WDM are summarized in eAppendix 1 in the Supplement.

A WDM with a lifetime horizon was implemented using discrete event simulation in SIMUL8 2019 software (Simul8 Corp). Discrete event simulation is an individual-level modeling approach in which the clinical course of individual patients through the system is determined according to their characteristics, previous events, and chance. The probability of events may be based on patient history (eg, number of previous relapses) within the model and demographic characteristics (eg, age and sex). The implemented WDM covers 16 components (Figure), grouped into the following 4 modules: module A, initial assessment pathway; module B, CHR pathway; module C, psychosis pathway; and module D, out-of-scope and death pathway. The overall model logic is described in detail in eAppendix 2 in the Supplement. A list of key assumptions and simplifications of the model is presented in eAppendix 3 in the Supplement.

**Figure.**
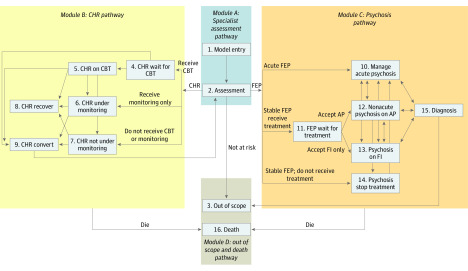
Model Structure Abbreviations: AP, antipsychotic medication; CBT, cognitive behavioral therapy; CHR, clinical high risk of psychosis; FEP, first-episode psychosis; and FI, family intervention.

Under the traditional piecewise framework for economic evaluation, the assessment of each decision problem listed in Table 1 would only involve running 1 part of the entire WDM (eg, assessment of topic 1 would only involve running the CHR pathway). However, because the underlying rationale of using a WDM approach is that all interventions are interrelated (ie, changes made to 1 intervention might affect the others), the entire WDM was run repeatedly for each decision problems listed in Table 1.

#### Input Data

In general, health economic models require 4 types of evidence, as follows: clinical evidence (baseline event risks, treatment effects and adverse events); health-related quality of life estimates (preference-based utility values); health care resource use; and costs. Model parameters were informed by numerous evidence sources, including systematic reviews and meta-analyses, clinical trials, clinical audits, observational studies, resource use surveys, costing studies, health valuation studies, and expert opinion. Evidence was mainly obtained from the meta-analyses conducted by the NICE schizophrenia Guideline Development Groups,^7,8^ supplemented with new evidence identified from rapid reviews of the literature conducted by the authors. A lack of evidence was identified for many parameters, including the long-term clinical effectiveness and adverse effects of interventions (eg, CBT, family intervention, and antipsychotic medications) and up-to-date costs of managing patients with schizophrenia in remission. When no relevant data were identified, expert opinion was used to inform the required model parameters, with alternative plausible values tested in sensitivity analyses. A summary of the key model parameters is reported in Table 2; a complete list of all model parameters, including data sources, is reported in eAppendix 4 in the Supplement.

**Table 2.** Summary of Key Parameters Used in the Schizophrenia Whole-Disease Model^a^

Parameter	Baseline value	Distribution
**Epidemiologic data, %**		
Age, mean, y	23.52	Normal (SE, 2.85)
Men	60.40	Beta (α = 665.61; β = 436.39)
Starting disease status		
Not at risk of psychosis	33.21	Dirichlet (n = 276, N = 831)
At CHR-P	34.90	Dirichlet (n = 290; N = 831)
FEP	31.89	Dirichlet (n = 265; N = 831)
**Service provision data, %**		
CBT		
Provision	41.01	Beta (α = 1011; β = 1454)
Take up	51.00	Beta (α = 510; β = 490)
Family intervention		
Provision	30.98	Beta (α = 589; β = 1312)
Take up	38.49	Beta (α = 224; β = 358)
Antipsychotic		
Provision	100.00	Assumed fixed
Take up for patients with FEP	97.38	Beta (α = 484; β = 13)
Delay in initiation of clozapine, y	3.98	Gamma (α = 137.25; β = 0.023)
**Clinical effectiveness data: nonpharmacologic interventions, RR**		
Transition to psychosis for CBT vs practice as usual	0.41	Log normal (ln[SE], 0.29)
Relapse for family intervention vs standard care or other control	0.63	Log normal (ln[SE], 0.16)
**Clinical effectiveness data: antipsychotic medication for individuals with FEP, OR**		
Annual probability of all-cause discontinuation for patients on placebo	0.82	Beta (α = 4949.54; β = 1079.87)
Amisulpride vs placebo	0.18	Log normal (ln[SE], 0.49)
Aripiprazole vs placebo	0.24	Log normal (ln[SE], 0.51)
Haloperidol vs placebo	0.21	Log normal (ln[SE], 0.34)
Olanzapine vs placebo	0.11	Log normal (ln[SE], 0.31)
Quetiapine vs placebo	0.21	Log normal (ln[SE], 0.32)
Risperidone vs placebo	0.15	Log normal (ln[SE], 0.40)
Haloperidol LAI vs placebo	0.15	Log normal (ln[SE], 0.45)
Paliperidone LAI vs placebo	0.19	Log normal (ln[SE], 0.53)
**Clinical effectiveness data: antipsychotics for individuals with TRS, OR**		
Annual probability of discontinuing clozapine because of inefficacy	0.02	Beta (α = 4.98; β = 310.02)
Haloperidol vs clozapine	5.56	Log normal (ln[SE], 0.35)
Olanzapine vs clozapine	1.37	Log normal (ln[SE], 0.34)
Quetiapine vs clozapine	4.35	Log normal (ln[SE], 0.69)
Risperidone vs clozapine	2.27	Log normal (ln[SE], 0.40)
**Health-related quality of life data**		
Individuals		
At CHR-P	0.71	Beta (α = 100.22; β = 40.78)
With psychosis in remission	0.80	Normal (SE, 0.04)
With psychosis in relapse	0.67	Normal (SE, 0.06)
Disutility		
Weight gain	0.03	Normal (SE, 0.01)
EPS	0.07	Normal (SE, 0.01)
Diabetes	0.09	Normal (SE, 0.05)
**Cost data, £**^b^		
CBT		
Cost per session	97.00	Gamma (α = 44.44; β = 2.18)
Sessions, No.	16	Assumed fixed
Family intervention		
Cost per session	112.00	Gamma (α = 44.44; β = 2.52)
Sessions, No.	20	Assumed fixed
Oral antipsychotic, per d		
Amisulpride	0.47	Gamma (α = 22.68; β = 0.02)
Aripiprazole	4.08	Gamma (α = 23.80; β = 0.17)
Haloperidol	0.37	Gamma (α = 30.86; β = 0.01)
Olanzapine	0.13	Gamma (α = 13.72; β = 0.01)
Quetiapine	1.24	Gamma (α = 6.25; β = 0.20)
Risperidone	0.36	Gamma (α = 5.41; β = 0.07)
Clozapine	1.56	Gamma (α = 156.25; β = 0.01)
LAI antipsychotic		
Haloperidol, 28 d	6.56	Gamma (α = 13.72; β = 0.48)
Paliperidone, 30 d	334.45	Gamma (α = 82.64; β = 4.05)
Attendance at clozapine clinic	16.40	Gamma (α = 44.44; β = 0.37)
Managing patients with nonrelapsed schizophrenia, per y	14 983.45	Gamma (α = 2.04; β = 7341.89)
Assessing an acute episode of psychosis	507.00	Gamma (α = 348.55; β = 1.45)
CRHT team, per contact	197.45	Gamma (α = 44.44; β = 4.44)
Contacts with CRHT team, mean, No.	16.3	Gamma (α = 78.32; β = 0.21)
Hospital bed-day	379.00	Gamma (α = 44.44; β = 8.52)
Bed-days during 1 relapse, mean, No.	138.90	Weibull (α = 0.65; β = 0.61)
Cost of adverse events		
Weight gain		
Year 1	97.20	Gamma (α = 44.44; β = 2.19)
Year 2 onwards	309.68	Gamma (α = 3.77; β = 6755.56)
Acute EPS, per episode	51.95	Gamma (α = 44.44; β = 1.17)
Diabetes, per y	1336.31	Gamma (α = 124 044.44; β = 0.01)
Neutropenia, per episode	469.48	Gamma (α = 92 802.96; β = 0.01)

Abbreviations: CBT, cognitive behavioral therapy; CHR-P, clinical high risk of psychosis; CRHT, crisis resolution and home treatment team; EPS, extrapyramidal symptoms; FEP, first-episode psychosis; LAI, long-acting injectable; ln, natural log; OR, odds ratio; RR, relative risk; TRS, treatment-resistant schizophrenia.

^a^ A complete list of all parameters used in the model and their data sources are reported in eAppendix 3 in the Supplement.

^b^ To convert to US dollars, multiply by 1.2776.

#### Model Checking

Extensive model verification and validation activities were undertaken, including white-box tests (scrutinizing the programming code) and black-box tests (testing the behavior of the model),^9^ checking results with stakeholders and comparing results with published literature. The details of white-box and black-box tests conducted are reported in eAppendix 5 in the Supplement. The overall model behavior was checked by 1 of us (P.T.). In addition, 7 members of a service user advisory group affiliated with the Maudsley Biomedical Research Centre, London, commented on the model structure and key assumptions.

#### Model Evaluation Methods

In accordance with the lower end of the cost-effectiveness threshold range used by NICE, interventions with an incremental cost-effectiveness ratio lower than £20 000 ($25 552) per QALY were considered cost-effective.^1^ The incremental cost-effectiveness ratio was defined as the difference in the expected cost of 2 interventions, divided by the difference in the expected effects of the 2 interventions.

Extensive sensitivity analyses were undertaken to test the robustness of the results of the base case analyses to different sets of assumptions and using different input data, including 1-way and multiway sensitivity analysis to assess the consequences of uncertainty regarding the value of a single or multiple parameter(s); structural sensitivity analysis to assess the consequences of uncertainty regarding the structural assumptions of the model (eg, whether CBT can prevent psychosis or just delay the transition to psychosis); and probabilistic sensitivity analyses that examine the consequences of joint uncertainty of multiple parameters simultaneously.

Following published guidance,^10^ a cohort of 200 000 patients was adopted for deterministic analyses and 1000 samples were used for probabilistic sensitivity analysis. The stability of results was tested to different numbers of patients and probabilistic sensitivity analysis runs. No prespecified level of statistical significance was set.

## Results

The base case and probabilistic sensitivity analysis results are presented in Table 3 and summarized in this section. The simulated cohort had a mean (SD) age of 23.5 (5.1) years, with 120 800 (60.4%) men, 66 400 (33.2%) not at risk of psychosis, 69 800 (34.9%) at CHR-P, and 63 800 (31.9%) with psychosis.

**Table 3.** Deterministic and Probabilistic Results of Cost-effectiveness Analysis

Intervention	Deterministic results					PSA results, per QALY^a^	
	Discounted mean		Incremental		ICER	WTP, £20 000^b^	WTP, £30 000^b^
	Cost per person, £^b^	QALYs per person	Cost, £^b^	QALY			
**Interventions for patients at CHR-P**							
PAU plus CBT	167 452	19.1904	−1243	0.0000	Dominating	0.95	0.95
PAU alone	168 695	19.1904	NA	NA	Dominated	0.05	0.05
**Interventions for individuals with acute psychosis**							
Mix of hospital admission and CRHT	168 078	19.1904	−3655	0.000	Dominating	1.00	1.00
Hospital admission alone	171 733	19.1904	NA	NA	Dominated	0.00	0.00
**First-line oral antipsychotic medication for FEP**							
Quetiapine	168 539	19.2005	1670	0.0071	235 211	0.06	0.06
Haloperidol	168 538	19.1981	NA	NA	Extendedly dominated	0.06	0.06
Aripiprazole	171 340	19.1977	NA	NA	Dominated	0.01	0.02
Risperidone	166 869	19.1934	1056	0.0112	94 286	0.30	0.30
Placebo	174 128	19.1931	NA	NA	Dominated	0.00	0.00
Amisulpride	165 813	19.1822	NA	NA	NA	0.39	0.39
Olanzapine	167 455	19.1794	NA	NA	Dominated	0.17	0.17
**Family intervention for FEP**							
Antipsychotic medication plus family intervention	167 905	19.2033	NA	NA	Dominating	0.58	0.62
Family intervention alone	175 065	19.1987	NA	NA	Dominated	0.09	0.10
Antipsychotic medication alone	168 261	19.1849	NA	NA	Dominated	0.33	0.28
**First-line oral antipsychotic medication for TRS**							
Clozapine	162 215	19.1977	NA	NA	Dominating	0.81	0.81
Olanzapine	165 444	19.1925	NA	NA	Dominated	0.16	0.16
Risperidone	169 324	19.1889	NA	NA	Dominated	0.03	0.03
Haloperidol	170 008	19.1883	NA	NA	Dominated	0.00	0.01
Quetiapine	172 043	19.1867	NA	NA	Dominated	0.00	0.00

Abbreviations: CBT, cognitive behavioral therapy; CHR-P, clinical high risk of psychosis; CRHT, crisis resolution and home treatment team; FEP, first-episode psychosis; ICER, incremental cost-effectiveness ratio; NA, not applicable; PAU, practice as usual; PSA, probabilistic sensitivity analysis; QALY, quality-adjusted life-year; TRS, treatment-resistant schizophrenia; WTP, willingness to pay.

^a^ Probability for the intervention to be most cost-effective within each topic.

^b^ To convert to US dollars, multiply by 1.2776.

### Interventions for Patients at CHR-P

The base case analysis suggests that practice as usual plus CBT dominates practice as usual alone. The cost savings of CBT are substantial (£1243 [$1588] per person), likely because the evidence used to inform the WDM suggests that CBT can delay the transition from CHR-P to psychosis, and the treatment cost for individuals with psychosis or schizophrenia is much higher than the treatment cost for individuals at CHR-P. On the other hand, the QALY gains of CBT are marginal (5.19 × 10^−5^ per person), likely because evidence used in the WDM suggests that the utility for individuals at CHR-P is similar to individuals with psychosis. Assuming a willingness-to-pay (WTP) threshold of £20 000 ($25 552) per QALY gained, the probability that practice as usual plus CBT is cost-effective compared to practice as usual alone was estimated to be 0.96.

### Interventions for Individuals With Acute Psychosis

The base case analysis suggests that a mix of CRHT and hospital admission produces the same QALY gains and additional cost savings (£3655 [$4670] per person) than hospital admission alone. This is because the evidence used to inform the WDM suggests equivalent effectiveness between hospital admission and CRHT,^11^ and the cost of hospital admission is much higher than the cost of CRHT services. Assuming a WTP threshold of £20 000 ($25 552) per QALY gained, the probability that a mix of CRHT and hospital admission is cost-effective compared with hospital admission alone was estimated to be 0.99.

### First-Line Oral Antipsychotic Medication for Individuals With FEP

The base case analysis suggests that, of the 7 interventions assessed, amisulpride was the most cost-effective option, followed by risperidone and olanzapine. This is because the evidence used to inform the WDM suggests that these antipsychotic medications are associated with the lowest probability of all-cause drug discontinuation. Assuming a WTP threshold of £20 000 ($25 552) per QALY gained, amisulpride is most likely to be cost-effective (0.39), followed by risperidone (0.30) and olanzapine (0.17). The probability of any antipsychotic medication being the most cost-effective option was less than 0.05.

### Family Intervention for Individuals With FEP

The base case analysis suggests that antipsychotic medication plus family intervention dominates both antipsychotic alone and family intervention alone. This is because the evidence used to inform the WDM suggests that family intervention can prevent relapse of psychosis, and the cost of treating relapse is much higher than the cost of family intervention. Assuming a WTP threshold of £20 000 ($25 552) per QALY gained, the probability that antipsychotic medication plus family intervention is the most cost-effective option compared with medication or family intervention alone was estimated to be 0.58.

### First-Line Oral Antipsychotic Medications for Individuals With TRS

The base case analysis suggests that clozapine dominates all other antipsychotic medications. This is because the evidence used in the WDM suggests that, of the 5 antipsychotics assessed for individuals with TRS, clozapine was associated with the lowest all-cause discontinuation rate (including discontinuation due to inefficacy, intolerability, and nonadherence) and was less likely to cause acute extrapyramidal symptoms. Assuming a WTP threshold of £20 000 ($25 552) per QALY gained, the probability that clozapine is the most cost-effective option compared with other medications was estimated to be 0.81.

### Summary of Base Case Results

Assuming a WTP pay threshold of £20 000 ($25 552) per QALY, the most cost-effective interventions were practice as usual plus 16 sessions of CBT for individuals with CHR-P. A mix of CRHT and hospital admission was most cost-effective for individuals with acute psychosis; amisulpride, risperidone, or olanzapine combined with 20 sessions of family intervention was most cost-effective for individuals with FEP; and clozapine was most cost-effective for individuals with TRS.

### Sensitivity Analyses

The results of the sensitivity analyses are summarized in eAppendix 6 in the Supplement. They suggest that the conclusions for interventions for patients at CHR-P, for individuals with acute psychosis, and for first-line oral antipsychotic medication for those with FEP and TRS were robust to all types of sensitivity analyses conducted. The conclusion for family intervention for individuals with FEP was robust to all types of sensitivity analyses except the following: changes in the choice of first-line antipsychotic; effectiveness of family intervention in preventing relapse; and number of family intervention sessions provided. Antipsychotic medication alone was the most cost-effective intervention when amisulpride was used as the first-line antipsychotic medications for individuals with FEP; when the relative risk of family intervention in preventing relapse was increased from 0.63 to 0.83; or when a brief (ie, number of sessions and effectiveness halved) version of family intervention was assumed.

## Discussion

### Comparing Results With Published Literature

Comparison of our results with published literature by individual topic is detailed in eAppendix 7 in the Supplement and summarized in this section. Our findings regarding interventions for patients at CHR-P and with acute psychosis and for first-line oral antipsychotic medication for individuals with FEP and with TRS are consistent with published literature.^12,13,14,15,16,17,18,19^ For first-line oral antipsychotic medication for individuals with FEP, both the schizophrenia WDM and the model developed by the NICE schizophrenia Guideline Development Group^7^ found that no antipsychotic medication can be considered clearly more cost-effective than the other options. However, the schizophrenia WDM found amisulpride to be the most cost-effective option, while the NICE model suggests amisulpride was the least cost-effective option. This is likely because of differences in the input data used. The systematic review conducted by the NICE schizophrenia Guideline Development Group in 2008^7^ found amisulpride to be associated with the second highest probability of relapse (second only to haloperidol), while the latest systematic reviews,^20,21^ which included additional trials, showed amisulpride to be associated with among the lowest probabilities of all-cause discontinuation and relapse rate.

### Implications for Clinical Practice

The results of our analyses suggest that adoption of the following interventions could result in cost savings compared with the current service: practice as usual plus 16 sessions of CBT for individuals at CHR-P; a mix of hospital admission and CRHT for individuals with acute psychosis; antipsychotic medication (amisulpride, risperidone, or olanzapine) combined with 20 sessions of family intervention for individuals with FEP; and clozapine for individuals with TRS. Adoption of clozapine for individuals with TRS also resulted in additional QALYs. The results suggest that a brief family intervention (ie, 10 sessions) would not be cost-effective for individuals with FEP.

### Strengths and Limitations

There are 3 key strengths of this study. First, it fills the evidence gap by presenting the first model-based economic analysis of the following interventions: CBT for individuals with CHR-P, CRHT for individuals with acute psychosis, family intervention for individuals with FEP, and clozapine and other atypical antipsychotics for individuals with TRS. There has been an increased interest in investment in brief family intervention because of resource constraints.^22^ However, our study showed that while a 20-session family intervention was cost-effective for individuals with FEP, a 10-session family intervention was not. This finding might change the current practice of providing family interventions. Second, this study provides an up-to-date assessment of antipsychotic medication for individuals with FEP based on the results of the latest network meta-analyses^20,21^ and modeled the cost and health consequences of 5 adverse effects of antipsychotics, including extrapyramidal symptoms, weight gain, glucose intolerance, diabetes, and neutropenia. Our analysis found amisulpride to be among the most cost-effective antipsychotics because of its therapeutic superiority in preventing relapse. Considering the current market share of amisulpride in the UK (ie, 1.39%),^23^ our findings may change the current clinical practice of prescribing antipsychotics. Third, all results presented within this study were based on a well-documented WDM, which is populated with input data carefully selected from high-quality literature. Extensive validation activities were undertaken to ensure the quality of the schizophrenia WDM. To our knowledge, this is the first WDM developed for the economic evaluation of a mental health disorder.

This study also has limitations. There are 2 major limitations of the schizophrenia WDM developed within this study. First, owing to resource constraints, input data for the WDM were obtained from published systematic reviews reported in the NICE schizophrenia guidelines,^7,8^ supplemented with new evidence identified from rapid reviews, rather than by undertaking our own de novo systematic reviews. As such, it is possible that newer high-quality evidence has not been included in the model. Second, as with any health economic model, the credibility of the schizophrenia WDM and its results are largely dependent on the quantity and quality of the evidence used to inform it. While searching for input data for the WDM, a lack of evidence was identified for many parameters, such as long-term clinical effectiveness and adverse effects of interventions (eg, CBT, family intervention, and antipsychotics) and up-to-date costs of managing patients with schizophrenia in remission. Even when evidence was available, it often had certain limitations, such as variation in criteria for relapse, relatively short follow-up periods, unclear masking, incomplete outcome data, and selective reporting. However, the model was designed to be adapted and reused, and thus, results can be updated as new or better-quality evidence is identified.

## Conclusions

The results of this study suggested that the following interventions are likely to be cost-effective: CBT for individuals at CHR-P; a mix of hospital admission and CRHT for individuals with acute psychosis; amisulpride, risperidone, or olanzapine combined with family intervention for individuals with FEP; and clozapine for individuals with TRS. Cost savings and additional quality-adjusted life-years may be gained by replacing current interventions with more cost-effective interventions.

## Notes

Supplement. **eAppendix 1. **Key Consequences of Interventions Considered in the Model **eAppendix 2. **Description of the Design-Oriented Model **eAppendix 3. **List of Assumptions and Simplifications of the Model **eAppendix 4. **Summary of Key Input Data **eAppendix 5. **White-Box and Black-Box Tests Conducted **eAppendix 6. **Results of Sensitivity Analysis **eAppendix 7. **Comparing Results With Published Literature **eReferences.**

## References

[1] National Institute for Health and Care Excellence Guide to the Methods of Technology Appraisal. National Institute for Health and Care Excellence; 2013. 27905712

[2] Jin H, Tappenden P, Robinson S, A systematic review of economic models across the entire schizophrenia pathway. Pharmacoeconomics. 2020. doi:10.1007/s40273-020-00895-6 32144726

[3] Tappenden P, Chilcott J, Brennan A, Squires H, Stevenson M Whole disease modeling to inform resource allocation decisions in cancer: a methodological framework. Value Health. 2012;15(8):1127-1136. doi:10.1016/j.jval.2012.07.008 23244816

[4] Jin H Using Whole Disease Modelling to Inform Resource Allocation Decisions in Schizophrenia Services. Thesis. King’s College London; 2019.

[5] Husereau D, Drummond M, Petrou S, Consolidated Health Economic Evaluation Reporting Standards (CHEERS) statement. Pharmacoeconomics. 2013;31(5):361-367. doi:10.1007/s40273-013-0032-y 23529207

[6] Fusar-Poli P, Byrne M, Badger S, Valmaggia LR, McGuire PK Outreach and support in south London (OASIS), 2001-2011: ten years of early diagnosis and treatment for young individuals at high clinical risk for psychosis. Eur Psychiatry. 2013;28(5):315-326. doi:10.1016/j.eurpsy.2012.08.002 23137782

[7] National Institute of Health and Care Excellence Psychosis and schizophrenia in adults: prevention and management. Updated March 1, 2014. Accessed April 29, 2020. https://www.nice.org.uk/guidance/cg178 32207892

[8] National Institute of Health and Care Excellence Psychosis and schizophrenia in children and young people: recognition and management. Updated October 26, 2016. Accessed April 29, 2020. https://www.nice.org.uk/guidance/cg155 32186837

[9] Pidd M Computer Simulation in Management Science. 5th ed John Wiley and Sons, Inc; 2006.

[10] Davis S, Stevenson M, Tappenden P, Wailoo AJ NICE DSU Technical Support Document 15: cost-effectiveness modelling using patient-level simulation. Published April 2014 Accessed September 9, 2018. http://nicedsu.org.uk/wp-content/uploads/2016/03/TSD15_Patient-level_simulation.pdf 27466644

[11] Murphy SM, Irving CB, Adams CE, Waqar M Crisis intervention for people with severe mental illnesses. Cochrane Database Syst Rev. 2015;(12):CD001087. doi:10.1002/14651858.CD001087.pub5 26633650 PMC7052814

[12] Ising HK, Lokkerbol J, Rietdijk J, Four-year cost-effectiveness of cognitive behavior therapy for preventing first-episode psychosis: the Dutch Early Detection Intervention Evaluation (EDIE-NL) Trial. Schizophr Bull. 2017;43(2):365-374. 27306315 10.1093/schbul/sbw084 PMC5605258

[13] McCrone P, Johnson S, Nolan F, Economic evaluation of a crisis resolution service: a randomised controlled trial. Epidemiol Psichiatr Soc. 2009;18(1):54-58. doi:10.1017/S1121189X00001469 19378700

[14] Gutierrez-Recacha P, Chisholm D, Haro JM, Salvador-Carulla L, Ayuso-Mateos JL Cost-effectiveness of different clinical interventions for reducing the burden of schizophrenia in Spain. Acta Psychiatr Scand Suppl. 2006;432(432):29-38. doi:10.1111/j.1600-0447.2006.00917.x 17087813

[15] Phanthunane P, Vos T, Whiteford H, Bertram M Cost-effectiveness of pharmacological and psychosocial interventions for schizophrenia. Cost Eff Resour Alloc. 2011;9(6):6. doi:10.1186/1478-7547-9-6 21569448 PMC3120770

[16] Anh NQ, Linh BN, Ha NT, Phanthunane P, Huong NT Schizophrenia interventions in Vietnam: primary results from a cost-effectiveness study. Glob Public Health. 2015;10(Supppl 1):S21-S39. doi:10.1080/17441692.2014.986158 25482499

[17] Davies LM, Drummond MF Assessment of costs and benefits of drug therapy for treatment-resistant schizophrenia in the United Kingdom. Br J Psychiatry. 1993;162:38-42. doi:10.1192/bjp.162.1.38 8425137

[18] Glennie J Pharmacoeconomic evaluations of clozapine in treatment-resistant schizophrenia and risperidone in chronic schizophrenia. Updated January 1, 1997. Accessed April 29, 2020. https://www.cadth.ca/pharmacoeconomic-evaluations-clozapine-treatment-resistant-schizophrenia-and-risperidone-chronic-0

[19] Oh PI, Iskedjian M, Addis A, Lanctôt K, Einarson TR Pharmacoeconomic evaluation of clozapine in treatment-resistant schizophrenia: a cost-utility analysis. Can J Clin Pharmacol. 2001;8(4):199-206. 11743592

[20] Zhao YJ, Lin L, Teng M, Long-term antipsychotic treatment in schizophrenia: systematic review and network meta-analysis of randomised controlled trials. BJPsych Open. 2016;2(1):59-66. doi:10.1192/bjpo.bp.115.002576 27703755 PMC4995551

[21] Leucht S, Cipriani A, Spineli L, Comparative efficacy and tolerability of 15 antipsychotic drugs in schizophrenia: a multiple-treatments meta-analysis. Lancet. 2013;382(9896):951-962. doi:10.1016/S0140-6736(13)60733-3 23810019

[22] Okpokoro U, Adams CE, Sampson S Family intervention (brief) for schizophrenia. Cochrane Database Syst Rev. 2014(3):CD009802. doi:10.1002/14651858.CD009802.pub2 24595545 PMC7437394

[23] NHS Digital Prescription Cost Analysis: England, 2016. Published March 30, 2017 Accessed March 3, 2016. https://digital.nhs.uk/data-and-information/publications/statistical/prescription-cost-analysis/prescription-cost-analysis-england-2016

