# Supplementary material for: Evaluation of the Cost-effectiveness of Services for Schizophrenia in the UK Across the Entire Care Pathway in a Single Whole-Disease Model
Source: JAMA Netw Open. 2020 May 27;3(5):e205888. doi: 10.1001/jamanetworkopen.2020.5888 (PMC7254180; doi:10.1001/jamanetworkopen.2020.5888)
Supplement: Supplement. — eAppendix 1. Key Consequences of Interventions Considered in the Model eAppendix 2. Description of the Design-Oriented Model eAppendix 3. List of Assumptions and Simplifications of the Model eAppendix 4. Summary of Key Input Data eAppendix 5. White-Box and Black-Box Tests Conducted eAppendix 6. Results of Sensitivity Analysis eAppendix 7. Comparing Results With Published Literature eReferences. [file jamanetwopen-3-e205888-s001.pdf]

Supplementary Online Content

Jin H, Tappenden P, MacCabe JH, Robinson S, Byford S. Evaluation of the cost-effectiveness of services for schizophrenia in the UK across the entire care pathway in a single whole-disease model. *JAMA Netw Open*. 2020;3(5):e205888. doi:10.1001/jamanetworkopen.2020.5888

eAppendix 1. Key Consequences of Interventions Considered in the Model

eAppendix 2. Description of the Design-Oriented Model

eAppendix 3. List of Assumptions and Simplifications of the Model

eAppendix 4. Summary of Key Input Data

eAppendix 5. White-Box and Black-Box Tests Conducted

eAppendix 6. Results of Sensitivity Analysis

eAppendix 7. Comparing Results With Published Literature

eReferences.

This supplementary material has been provided by the authors to give readers additional information about their work.

eAppendix 1. Key Consequences of Interventions Considered in the Model

The key impacts of interventions considered in the model are summarised in eTable 1. It should be noted that not all impacts of interventions were included; common reasons for exclusion are: (1) not expected to affect results; (2) lack of evidence.

© 2020 Jin H et al. *JAMA Network Open.*

eTable 1: Key impacts of interventions modelled in the schizophrenia WDM

	Clinical benefits	Clinical harms	Costs	Cost savings
Topic A – Interventions for patients at CHR				
**PAU**	Not explicitly modelled	None	Cost of providing PAU	Not explicitly modelled
**PAU + CBT**	Delaying transition from CHR to psychosis	None	Cost of providing PAU and CBT	Delaying the treatment costs of psychosis
Topic B – Interventions for people with acute psychosis				
**Hospital admission**	Not explicitly modelled	Not explicitly modelled	Cost of hospital admission	Not explicitly modelled
**A mix of CRHT and hospital admission**	Assumed to be the same as hospital admission	Assumed to be the same as hospital admission	Cost of hospital admission and contacts with CRHT	Reduction in the number of hospital admissions
Topic C – First-line oral antipsychotic medication for people with FEP				
**Different antipsychotics**	Preventing relapse of psychosis	Adverse events of antipsychotics, including acute EPS, weight gain, glucose intolerance, diabetes	Cost of antipsychotic and cost of treating adverse events	Reduced costs of treating relapse
Topic D – Family intervention for people with FEP				
**Family intervention alone**	Preventing relapse of psychosis	None	Cost of providing family intervention	Reducing the cost of treating relapse
**Antipsychotic alone**	Preventing relapse of psychosis	Adverse events of antipsychotics, including acute EPS, weight gain, glucose intolerance, diabetes	Cost of antipsychotic and cost of treating adverse events	Reducing the cost of treating relapse
**Antipsychotic + family intervention**	Preventing relapse of psychosis	Adverse events of antipsychotics, including acute EPS, weight gain, glucose intolerance, diabetes	Cost of antipsychotic, cost of treating adverse events of antipsychotics, and cost of providing family intervention	Reducing the cost of treating relapse
Topic E – First-line oral antipsychotic medication for people with treatment-resistant schizophrenia				
**Different antipsychotics**	Preventing relapse of psychosis	Adverse events of antipsychotics, including acute EPS, weight gain, glucose intolerance, diabetes and neutropenia (for patients taking clozapine)	Cost of antipsychotic and cost of treating adverse events	Reduced costs of treating relapse

Abbreviations:
CBT: cognitive behaviour therapy; CHR: clinical high risk of psychosis; CRHT: crisis resolution and home treatment team; EPS: extrapyramidal Symptoms; FEP: first episode psychosis; PAU: practice as usual.

© 2020 Jin H et al. *JAMA Network Open.*

eAppendix 2. Description of the Design-Oriented Model

A detailed description of each module within the "design-oriented" model is presented in the following order:

- Module A – Initial assessment pathway (Section 2.1)
- Module B – Clinical high risk of psychosis (CHR) pathway (Section 2.2)
- Module C – Psychosis pathway (Section 2.3)
- Module D – Out of scope and death pathway (Section 2.4)

This description should be read in conjunction with EFigure 1.

2.1 Module A – Initial assessment pathway

Patients enter the simulation model (Part 1 'Model entry') having been referred to secondary mental health care services, either by their GP, A&E, Early Intervention in Psychosis (EIP) service, school teachers, staff in the criminal justice sector, or family members, carers or neighbours of people with psychotic symptoms. After being assessed by a mental health specialist (Part 2 'Assessment'), patients are triaged to different treatment pathways based on their assessment results and interventions they are going to receive. It is assumed that a diagnosis of schizophrenia cannot be made or ruled out during patients' initial specialist assessment, as the diagnosis process can take six months or more. There are three possible results of the initial specialist assessment: not at risk of psychosis, CHR and FEP. Which intervention will a specific patient receive depends on three factors:

1) Assessment result. Patients assessed as being not at risk of psychosis receive no intervention. Patients assessed as CHR are eligible for CBT; while patients assessed as FEP are eligible for antipsychotic medications, CBT and family intervention.
2) Local availability of the intervention. It is assumed that despite the NICE recommendation, CBT and family interventions are not always provided by the local NHS trusts. Antipsychotic medications are assumed to be always available for patients with psychosis.
3) Patients' acceptability of interventions. In the model, it is assumed that:
    - the uptake of CBT and monitoring is optional for patients at CHR;
    - the uptake of family intervention is optional for patients with psychosis or schizophrenia;
    - the prescription of antipsychotic medications is optional for patients with a first episode of psychosis in a seTable condition, and is compulsory for all the other patients with psychosis (although patients might not adhere to the medication prescribed to them).

After receiving a specialist mental health assessment at Part 2 'Assessment', patients are triaged to different pathways:

- Patients assessed as CHR move to Module B 'CHR pathway';
- Patients assessed as FEP move to Module C 'Psychosis pathway';
- Patients deemed to be not at risk of psychosis move to Module D 'Out of scope and death pathway'.

2.2 Module B – CHR pathway

Patients can enter the CHR pathway through different routes depending on their acceptability of and access to interventions:

- Patients who agree to receive and have access to CBT move to Part 4 'CHR wait for CBT'. While waiting for CBT, patients can either convert to psychosis, or do not convert during the waiting period. The former group move to Part 9 'CHR convert', while the latter group move to Part 5 'CHR on CBT' to receive a full-course (16 sessions) of individual CBT. While patients are receiving CBT, they can convert to FEP, recover, or stay as CHR. Patients who convert to FEP stop receiving CBT and move to Part 9 'CHR convert'. Patients who achieve recovery are assumed to finish the full-course of CBT before moving to Part 8 'CHR recover'. Patients who have not achieved recovery after receiving a full-course of CBT, and who agree to receive and have access to monitoring move to Part 6 'CHR under monitoring'. Patients who have not achieved recovery after receiving a full-course of CBT, but who do not agree to receive or do not have access to monitoring move to Part 7 'CHR not under monitoring'.
- Patients who do not agree to receive CBT or do not have access to CBT but agree to receive monitoring and have access to monitoring move to Part 6 'CHR under monitoring'. Those patients will be monitored for up to three years. During the three years, patients can either convert to psychosis or recover. Patients who convert move to Part 9 'CHR convert', and then

© 2020 Jin H et al. *JAMA Network Open.*

move to Part 2 ‘Assessment’ to receive another specialist assessment; while patients who recover move to Part 8 ‘CHR recover’. For the sake of simplicity, it is assumed that recovered CHRs cannot relapse or go on to develop psychosis.
- Patients who do not agree to receive any interventions (CBT or monitoring) or do not have access to any interventions move to Part 7 ‘CHR not under monitoring’. While staying at this workcentre, patients can either convert to psychosis or recover. The former group move to Part 9 ‘CHR convert’, while the latter group move to Part 8 ‘CHR recover’.

Patients at any part of the CHR pathway are at risk of death. Patients who die move to Module D ‘Out of scope and death pathway’. For the sake of simplicity, the risk of dying is not repeated below when describing the logic for every single part of the pathway.

2.3 Module C – Psychosis pathway

Patients can enter the psychosis pathway through different routes depending on their disease status, and the acceptability of and access to interventions:

- Patients who are assessed as acute FEP move to Part 10 ‘Manage acute psychosis’, where they will receive antipsychotic medications and other acute treatments without delay. Patients at Part 10 can experience different events: due for the next family intervention session (if the patients are receiving family intervention), receive a formal diagnosis, and achieve remission. Depending on which of the above events happen first, patients move to different parts. Patients who are due for the next family intervention session move to Part 13 ‘Psychosis on family intervention’. Once a family intervention session is finished, patients return to Part 10 ‘Manage acute psychosis’. Patients who receive a formal diagnosis move to Part 15 ‘Diagnosis’: if the diagnosis result is schizophrenia, patients return to Part 10 ‘Manage acute psychosis’ to continue their current treatments; if the diagnosis result is not schizophrenia, patients move to Part 3 ‘Out of scope’. Patients who achieve remission move to Part 12 ‘Non-acute psychosis on antipsychotics’ to receive treatment for seTable patients.
- Patients who are assessed as seTable FEP, and who have access to and agree to receive interventions move to Part 11 ‘FEP wait for treatment’. Those patients are assumed to experience a delay before receiving treatment. When the waiting time finishes, patients who accept antipsychotic medication with or without family intervention move to Part 12 ‘Non-acute psychosis on antipsychotic medication’, while patients who only accept family intervention move to Part 13 ‘Psychosis on family intervention’. Patients at Part 12 ‘Non-acute psychosis on antipsychotic medication’ can experience different events: due for the next family intervention session, receive a formal diagnosis, and discontinue antipsychotic due to inefficacy, intolerability or non-adherence. Depending on which of the above events happen first, patients move to different parts of the model as follows:
  - Patients who are due for the next family session move to Part 13 ‘Psychosis on family intervention’. Once a family intervention session is finished, patients return to Part 12 ‘Non-acute psychosis on antipsychotic medication’.
  - Patients who receive a formal diagnosis move to Part 15 ‘Diagnosis’: if the diagnosis result is schizophrenia, patients return to Part 11 ‘Non-acute psychosis on antipsychotics’ to continue their current treatments; if the diagnosis result is not schizophrenia, patients move to Part 3 ‘Out of scope’.
  - Patients who discontinue their antipsychotic medication due to inefficacy are assumed to experience a relapse and move to Part 10 ‘Manage acute psychosis’.
  - Patients who discontinue their antipsychotic medication due to intolerability are assumed to be switched to another antipsychotic medication and stay at Part 12 ‘Non-acute psychosis on antipsychotic medication’.
  - Patients who discontinue their antipsychotics due to non-adherence move to Part 14 ‘Psychosis stop treatment’.
- Patients who are assessed as seTable FEP and who choose not to have antipsychotic medication or family intervention due to refusal or local unavailability move to Part 14 ‘Psychosis stop treatment’. Those patients are assumed to receive no intervention and stay at the current part until they experience a relapse, then they will move to Part 10 ‘Manage acute psychosis’.

Patients at any part of the psychosis pathway are at risk of death. Patients who die move to Module D ‘Out of scope and death pathway’.

2.4 Module D – Out of scope and death pathway

© 2020 Jin H et al. *JAMA Network Open.*

Patients who are not at risk of psychosis or receive a non-schizophrenia diagnosis move to Part 3 'Out of scope'. For the sake of simplicity, it is assumed that patients on Part 3 cannot go on to develop psychosis. Patients who die move to Part 16 'Death', where their life-time costs and QALYs are calculated separately by aggregating adding together the cost and QALYs that each patient has accrued at each part in the model that they passed through prior to death. Patients' costs at each part in the model were determined by the interventions that they received, including cost of providing the interventions, and cost of treating adverse events (weight gain, EPS, diabetes and neutropenia). Patients' utility at different parts in the model were determined based on their age, gender, disease status (not at risk of psychosis, CHR or psychosis) and presence of adverse events (weight gain, EPS and diabetes). Patients' life expectancies were determined based on their age, gender, disease status and intervention received (e.g. whether clozapine was used for people with TRS).

© 2020 Jin H et al. *JAMA Network Open.*

eAppendix 3. List of Assumptions and Simplifications of the Model

3.1 Key assumptions of the model

- A DUP longer than 6 months is associated with poor prognosis, compared with a DUP less than 6 months
- Patients can achieve clinical recovery without antipsychotic medications
- Psychotic patients who have achieved clinical recovery while on antipsychotic medication will adhere to their current antipsychotic medication

3.2 Key simplifications of the model

- Impacts of treatment for co-existing conditions was not explicitly modelled
- The mental health status for people with psychosis or schizophrenia were grouped into two categories: relapse and remission
- The sensitivity and specificity of a specialist assessment is assumed to be 100%
- Patients who are assessed as not at risk of psychosis during mental health specialist assessment cannot go on to develop psychosis
- CHRs who have access to and accept CBT will finish a full course of CBT (16 sessions), unless they die or convert to psychosis before that
- Recovered CHRs cannot relapse or go on to develop psychosis
- Services for patients with psychosis or schizophrenia were grouped into two categories: services for acute patients and services for non-acute patients
- Reasons for discontinuing antipsychotic medication were grouped into three mutually exclusive categories: inefficacy, intolerance and non-adherence
- Dosing effects of antipsychotic medication were not modelled
- Patients with psychosis or schizophrenia who achieved clinical recovery are no longer at risk of relapse

© 2020 Jin H et al. *JAMA Network Open.*

eAppendix 4. Summary of Key Input Data

This section presents a summary of the key input data used in the WDM. The input data are reported in the following order:

- Epidemiological data (Section 3.1)
- Service provision data (Section 3.2)
- Clinical effectiveness data (Section 3.3)
- Health-related quality of life (HRQoL) data (Section 3.4)
- Resource use and unit cost data (Section 3.5)

4.1 Epidemiological data

eTable 2: Summary of key epidemiological data

Parameter	Mean	Distribution	Source
Demographic details			
Mean age	23.52 y	Normal (SE=2.85)	[1]
Male	60.40%	Beta (α=665.61; β=436.39)	(Fusar-Poli *et al*., 2013)
Disease status			
Not at risk of psychosis	33.21%	Dirichlet (n=276)	[1]
CHR	34.90%	Dirichlet (n=290)	(Fusar-Poli *et al*., 2013)
FEP	31.89%	Dirichlet (n=265)	(Fusar-Poli *et al*., 2013)
Proportion of FEP in need of acute care	43.09%	Beta (α=183.61; β=243.39)	[2]
Proportion of FEP who eventually receive a diagnosis of schizophrenia	71.58%	Beta (α=277.00; β=110.00)	[3]
Proportion of patients with a short DUP (<6 months) for patients with FEP			
Short DUP (<6 months)	66.56%	Beta (α=228.00; β=115.00)	[4]
Duration of DUP for patients with a short DUP (<6 months)			
Delay in help-seeking	12.70 days	Gamma (α=47.24; β=0.27)	[4]
Delay in referral to mental health services	8.20 days	Gamma (α=5.01; β=1.64)	(Birchwood *et al*., 2013
Duration of DUP for patients with a short DUP (≥6 months)			
Delay in help-seeking	254.60 days	Gamma (α=40.37; β=6.31)	(Birchwood *et al*., 2013
Delay in referral to mental health services	157.00 days	Gamma (α=20.06; β=7.83)	(Birchwood *et al*., 2013
Prognosis for CHR			
Probability of converting to FEP	0.27	Beta (α=664.52; β=1,837.48)	[5]
Time to different prognosis outcomes for CHR			
Time to convert to FEP	1.33 years	Weibull (α=1.44; β=1.46)	[5]
Time to recovery	1.19 years	Weibull (α=1.64; β=1.34)	[5]
Prognosis for FEP			
Probability of recovery for FEP with short DUP (<6 months)	0.27	Beta (α=37.00; β=101.00)	[6]
Relative risk of recovery (long DUP vs short DUP)	0.25	Log normal (ln(SE)=0.35)	[6]
Time to first remission for FEP			
Time to first remission	0.89 years	Weibull (α=0.62; β=0.44)	[6]
Time to recovery after achieving first remission	2.00 years	Assumed fixed	[7]
Life expectancy for people not at risk of psychosis or at CHR			
Age and sex specific life expectancy	Obtained from the UK national life eTable		[8]
Standard mortality ratio (SMR) for people with psychosis/schizophrenia			
SMR (age 16-29 years)	7.40	Log normal (ln(SE)=0.38)	[3]
SMR (age 30-44 years)	5.80	Log normal (ln(SE)=0.23)	[3]
SMR (age 45-59 years)	2.50	Log normal (ln(SE)=0.36)	[3]
SMR (age 60-74 years)	1.70	Log normal (ln(SE)=0.40)	[3]

Abbreviations:

CHR: clinical high risk of psychosis; DUP: duration of untreated psychosis; FEP: first episode psychosis; PAU: practice as usual; SE: standard errors.

4.2 Service provision data

eTable 3: Summary of key service provision data

Parameter	Mean	Distribution	Source
Waiting time for patients at CHR *(Data used in Module B – 'CHR pathway')*			
Waiting time for CHR to be accepted by the CBT provider	30.00 days	Exponential	Expert opinion
Waiting time for specialist assessment for converted CHR	14.00 days	Exponential	[9]
Waiting time for people with FEP *(Data used in Module C – 'Psychosis pathway')*			
Waiting time for interventions (short DUP <6 months)	15.70 days	Gamma (α=106.32; β=0.15)	[4]
Waiting time for interventions (Long DUP ≥6 months)	292.60 days	Gamma (α=126.35; β=2.32)	(Birchwood *et al*., 2013)
Waiting time for diagnosis	0.66 years	Weibull (α=0.62; β=0.44)	[10]
Waiting time for treatment-resistant schizophrenia (TRS) *(Data used in Module C – 'Psychosis pathway')*			
Delay in initiation of clozapine	3.98 years	Gamma (α=137.25; β=0.023)	[11]
Provision and take up of CBT for people at CHR			
Provision of CBT	41.01%	Beta (α=1,011; β=1,454)	[12]
Take up of CBT	51.00%	Beta (α=510; β=490)	[12]
Provision and take up of monitoring for people at CHR			
Provision of monitoring	90.00%	Beta (α=180; β=20)	Expert opinion
Take up of monitoring	78.79%	Beta (α=338; β=91)	[13]
Provision and take up of family intervention for people with psychosis/schizophrenia			
Provision of family intervention	30.98%	Beta (α=589; β=1,312)	[12]
Take up of family intervention	38.49%	Beta (α=224; β=358)	[12]
Provision and take up of antipsychotic medication for people with psychosis/schizophrenia			
Provision of antipsychotic medication	100.00%	Assume fixed	Expert opinion
Take up of antipsychotic medication for patients with FEP	97.38%	Beta (α=484; β=13)	[2]
Take up of antipsychotic medication for patients with relapsed psychosis/ schizophrenia	It was assumed that all patients with relapsed psychosis will be prescribed with antipsychotic medication and will take the medication (although they may discontinue their antipsychotic medication due to non-adherence)		
Market share of 1st line oral antipsychotic medications for patients with FEP			
Aripiprazole	13.30%	Dirichlet (n=61)	[2]
Olanzapine	40.20%	Dirichlet (n=185)	[2]
Quetiapine	25.20%	Dirichlet (n=116)	[2]
Risperidone	19.10%	Dirichlet (n=88)	[2]
Amisulpride	1.39%	Dirichlet (n=6)	Calculated based on data reported by Prescription Cost Analysis [14]
Haloperidol	0.81%	Dirichlet (n=4)	
Market share of oral antipsychotic medications for patients with relapsed psychosis			
Aripiprazole	12.27%	Dirichlet (n^1=14,280,763)	Calculated based on data reported by Prescription Cost Analysis [14]
Olanzapine	38.48%	Dirichlet (n^1=44,786,709)	
Quetiapine	26.11%	Dirichlet (n^1=30,391,781)	
Risperidone	14.34%	Dirichlet (n^1=16,687,517)	
Amisulpride	5.55%	Dirichlet (n^1=6,463,921)	
Haloperidol	3.25%	Dirichlet (n^1=3,781,443)	
Market share of long-acting injeceTable (LAI) antipsychotic medication for patients who have history of non-adherence			
Haloperidol LAI	97.37%	Dirichlet (n^1=9,738,680)	Calculated based on data reported by Prescription Cost Analysis [14]
Paliperidone LAI	2.63%	Dirichlet (n^1=262,890)	

Abbreviations:
CHR: clinical high risk of psychosis; DUP: duration of untreated psychosis; FEP: first episode psychosis; LAI: long-acting injeceTable; PAU: practice as usual; SE: standard errors.

© 2020 Jin H et al. *JAMA Network Open.*

4.3 Clinical effectiveness data

eTable 4: Summary of key clinical effectiveness data

Parameter	Mean	Distribution	Source
Clinical effectiveness of CBT for delaying/preventing psychosis			
RR of transition to psychosis (CBT vs usual or non-specific control treatment)	0.41	Log normal (ln(SE)=0.29)	[15]
Clinical effectiveness of family interventions for preventing relapse			
RR of relapse (family intervention vs standard care or other control)	0.63	Log normal (ln(SE)=0.16)	[16]
All-cause discontinuation of antipsychotic medications for psychotic patients who are not treatment resistant			
Annual probability of all-cause discontinuation for patients on nothing/placebo	0.82	Beta (α=4,949.54; β=1079.87)	[16]
OR Amisulpride vs placebo	0.18	Log normal (ln(SE)=0.49)	[17]
OR Aripiprazole vs placebo	0.24	Log normal (ln(SE)=0.51)	[17]
OR Haloperidol vs placebo	0.21	Log normal (ln(SE)=0.34)	[17]
OR Olanzapine vs placebo	0.11	Log normal (ln(SE)=0.31)	[17]
OR Quetiapine vs placebo	0.21	Log normal (ln(SE)=0.32)	[17]
OR Risperidone vs placebo	0.15	Log normal (ln(SE)=0.40)	[17]
OR Haloperidol LAI vs placebo	0.15	Log normal (ln(SE)=0.45)	[17]
OR Paliperidone LAI vs placebo	0.19	Log normal (ln(SE)=0.53)	[17]
Discontinuation of antipsychotic medications for patients with TRS due to inefficacy			
Annual probability of discontinuing clozapine due to inefficacy	0.02	Beta (α=4.98; β=310.02)	[18]
OR Haloperidol vs clozapine	5.56	Log normal (ln(SE)= 0.35)	[19]
OR Olanzapine vs clozapine	1.37	Log normal (ln(SE)=0.34)	[19]
OR Quetiapine vs clozapine	4.35	Log normal (ln(SE)=0.69)	[19]
OR Risperidone vs clozapine	2.27	Log normal (ln(SE)=0.40)	[19]
Adverse events of antipsychotic medications – Weight gain			
Annual probability of weight gain for patients on placebo	0.13	Beta (α=45.98; β=307.70)	[20]
OR Amisulpride vs placebo	1.85	Log normal (ln(SE)= 0.60)	[17]
OR Aripiprazole vs placebo	1.53	Log normal (ln(SE)=0.57)	[17]
OR Haloperidol vs placebo	1.03	Log normal (ln(SE)=0.52)	[17]
OR Olanzapine vs placebo	4.27	Log normal (ln(SE)=0.37)	[17]
OR Quetiapine vs placebo	1.71	Log normal (ln(SE)=0.43)	[17]
OR Risperidone vs placebo	1.87	Log normal (ln(SE)=0.63)	[17]
OR Haloperidol LAI vs placebo	1.18	Log normal (ln(SE)=0.77)	[17]
OR Paliperidone LAI vs placebo	2.06	Log normal (ln(SE)=0.62)	[17]
RR Clozapine vs haloperidol	1.28	Log normal (ln(SE)=0.09)	[21]
Adverse events of antipsychotic medications – EPS			
Annual probability of EPS for patients on placebo	0.11	Beta (α=103.32; β=835.92)	[20]
OR Amisulpride vs placebo	0.99	Log normal (ln(SE)=0.35)	[17]
OR Aripiprazole vs placebo	0.87	Log normal (ln(SE)=0.42)	[17]
OR Haloperidol vs placebo	2.34	Log normal (ln(SE)=0.31)	[17]
OR Olanzapine vs placebo	0.51	Log normal (ln(SE)=0.34)	[17]
OR Quetiapine vs placebo	0.69	Log normal (ln(SE)=0.30)	[17]
OR Risperidone vs placebo	0.96	Log normal (ln(SE)=0.96)	[17]
OR Haloperidol LAI vs placebo	6.02	Log normal (ln(SE)=0.64)	[17]
OR Paliperidone LAI vs placebo	2.89	Log normal (ln(SE)=0.57)	[17]
OR Clozapine vs haloperidol	0.07	Log normal (ln(SE)=0.88)	[19]
Adverse events of antipsychotic medications – Glucose intolerance			
Annual probability of developing glucose intolerance for patients on nothing/placebo	0.064	Beta (α=5.28; β=77.21)	[22]
OR Amisulpride vs placebo	0.46	Log normal (ln(SE)=0.25)	[17]
OR Aripiprazole vs placebo	0.29	Log normal (ln(SE)=0.17)	[23]
OR Haloperidol vs placebo	0.39	Log normal (ln(SE)=0.04)	[17]
OR Olanzapine vs placebo	0.76	Log normal (ln(SE)=0.03)	[17]
OR Quetiapine vs placebo	0.49	Log normal (ln(SE)=0.08)	[17]
OR Risperidone vs placebo	0.46	Log normal (ln(SE)=0.04)	[23]
OR Haloperidol LAI vs placebo	0.39	Log normal (ln(SE)=0.87)	[17]
OR Paliperidone LAI vs placebo	3.06	Log normal (ln(SE)=0.82)	[17]
RR Clozapine vs olanzapine	1.29	Log normal (ln(SE)=0.35)	[24]
Transition from glucose intolerance to diabetes			
Annual transition probability	0.02	Beta (α=60.24; β=3013.29)	[25]
Adverse events of antipsychotic medications – Diabetes			
Annual probability of developing diabetes for patients on nothing/placebo	0.021	Beta (α=3.57; β=166.29)	[22]
Adverse events of antipsychotic medications – Neutropenia (for patients on clozapine only)			
Annual probability of developing neutropenia	0.02	Beta (α=7.57; β=307.43)	[18]

Abbreviations:
CBT: cognitive behaviour therapy; CHR: clinical high risk of psychosis; DUP: duration of untreated psychosis; EPS: extrapyramidal symptoms; FEP: first episode psychosis; LAI: long-acting injeceTable; PAU: practice as usual; OR: odds ratio; SE: standard errors.

© 2020 Jin H et al. *JAMA Network Open.*

4.4 HRQoL data

eTable 5: Estimated general population HRQoL values[1]

	Utility value (mean)	
**Age**	**Male**	**Female**
20	0.954	0.932
5	0.945	0.924
30	0.934	0.913
35	0.922	0.901
40	0.909	0.887
45	0.893	0.872
50	0.876	0.855
55	0.857	0.836
60	0.837	0.816
65	0.815	0.794
70	0.791	0.770
75	0.766	0.745
80	0.739	0.718
85	0.710	0.689
90	0.680	0.659

Notes:
1. Based on the regression model by Ara & Brazier [26].

eTable 6: Summary of key HRQoL data

	Utility	Distribution	Source
HRQoL data for people at different stages of schizophrenia			
People at CHR	0.71	Beta (α=100.22; β=40.78)	Multiplicative utility calculated from [27]
People with untreated FEP	0.59	Beta (α=62.43; β=42.57)	Multiplicative utility calculated from [27]
People with psychosis in remission	0.80	Normal (SE=0.04)	Derived from Lenert *et al.* [28]
People with psychosis in relapse	0.67	Normal (SE=0.06)	
Disutility caused by adverse events of antipsychotic medication			
Weight gain	0.03	Normal (SE=0.01)	Derived from Lenert *et al.* [28]
EPS	0.07	Normal (SE=0.01)	
Diabetes	0.09	Normal (SE=0.05)	Calculated from Clarke *et al.* [29]

Abbreviations:
CHR: clinical high risk of psychosis; EPS: extrapyramidal symptoms; FEP: first episode psychosis; HRQoL: health-related quality of life; SE: standard errors.

© 2020 Jin H et al. *JAMA Network Open.*

4.5 Resource use and unit cost data

eTable 7: Summary of key resource use and unit cost data

Parameter	Mean	Distribution	Source
Cost of CBT			
Cost per session	£97.00	Gamma (α=44.44; β=2.18)	PSSRU [30]
No. of sessions	16	Assumed fixed	
**Total cost**	**£1,552.00**		
Cost of monitoring patients at CHR			
Telephone monitoring	£19.75	Gamma (α=44.44; β=0.44)	PSSRU [30]
Face-to-face monitoring	£79.00	Gamma (α=44.44; β=1.78)	PSSRU [30]
Cost of family intervention			
Cost per session	£112.00	Gamma (α=44.44; β=2.52)	PSSRU [30]
No. of sessions	20	Assumed fixed	
**Total cost**	**£2,240.00**		
Daily cost of oral antipsychotics			
Amisulpride	£0.47	Gamma (α= 22.68; β=0.02)	Prescription Cost Analysis [14]
Aripiprazole	£4.08	Gamma (α=23.80; β=0.17)	
Haloperidol	£0.37	Gamma (α=30.86; β=0.01)	
Olanzapine	£0.13	Gamma (α=13.72; β=0.01)	
Quetiapine	£1.24	Gamma (α=6.25; β=0.20)	
Risperidone	£0.36	Gamma (α=5.41; β=0.07)	
Clozapine	£1.56	Gamma (α=156.25; β=0.01)	
Cost of long-acting injeceTable (LAI) antipsychotics (per injection)			
Haloperidol LAI (28 days)	£6.56	Gamma (α=13.72; β=0.48)	Prescription Cost Analysis [14]
Paliperidone LAI (30 days)	£334.45	Gamma (α=82.64; β=4.05)	
Cost of LAI administration	£7.17	Gamma (α=44.44; β=0.16)	PSSRU [30]
Cost of neutrophil monitoring for patients on clozapine			
Blood test	£2.65	Gamma (α=44.44; β=0.06)	Uplifted from [31]
Attendance at clozapine clinic	£16.40	Gamma (α=44.44; β=0.37)	PSSRU [30]
Cost of treating adverse events of antipsychotics			
Weight gain *(Year 1)*	£97.20 per year	Gamma (α=44.44; β=2.19)	PSSRU [30]
Weight gain *(Year 2 onwards)*	£309.68 per year	Gamma (α=3.77; β= 6,755.56)	Calculated from [32]
EPS	£51.95 per episode	Gamma (α=44.44; β=1.17)	PSSRU [30] and BNF [33]
Diabetes	£1,336.31 per year	Gamma (α=124,044.44; β=0.01)	[34]
Neutropenia	£469.48 per episode	Gamma (α=92,802.96; β=0.01)	NHS Reference Costs 2015-16 [35]
Cost of managing patients with seTable psychosis			
Annual cost of managing non-relapsed schizophrenia patients	£14,983.45	Gamma (α=2.04; β=7,341.89)	Uplifted from the NICE guideline [16]
Cost of assessing one acute episode of psychosis			
Cost of assessing an acute episode of psychosis	£507.00	Gamma (α= 348.55; β=1.45)	NHS Reference Costs 2015-16 [35]
Cost of managing one acute episode of psychosis at a CRHT			
Cost per contact with CRHT team	£197.45	Gamma (α=44.44; β=4.44)	Uplifted from the NHS Reference Costs 2012-2013 [36]
Average number of contacts with CRHT team	16.3	Gamma (α=78.32; β=0.21)	[37]
**Total cost**	**£3,218.44**		
Cost of managing one acute episode of psychosis in hospital			
Cost per bed day	£379.00	Gamma (α=44.44; β=8.52)	PSSRU [30]
Average number of bed days	138.90	Weibull (α=0.65; β=0.61)	[38]
**Total cost**	**£32,237.74**		
Proportion of patients with mental and behavioural disorders who die in hospital			
Hospital	28.70%	Beta (α=13866.00; β=34451.00)	[39]
Cost of patients who have died by setting			
Died in acute hospital	£3,222.84	Gamma (α=384.47; β=8.38)	Uplifted from [40]
Died in community health and social care	£2,215.67	Gamma (α=123.46; β=17.95)	

Abbreviations:
CBT: cognitive behaviour therapy; CHR: clinical high risk of psychosis; CRHT: crisis resolution and home treatment team; EPS: extrapyramidal symptoms; FEP: first episode psychosis; LAI: long-acting injeceTable; SE: standard errors.

© 2020 Jin H et al. *JAMA Network Open.*

eAppendix 5. White-Box and Black-Box Tests Conducted

5.1 White-box tests conducted

Extensive white-box tests were conducted to check the behaviour of model logic, including:

- Check the behaviour of individual workcentres. For example, the values of relevant attributes (e.g. patient's age and accumulated cost) were recorded when the patient entered and left a particular workcentre and checked against the patient's disease status, prognosis and treatment plans. For workcentres which involve complex calculations, the values of relevant attributes were recalculated in Excel and compared with the values produced by SIMUL8.
- Check the behaviour of multiple workcentres. For example, the interventions that patients received were recorded and compared against patient's attributes when entering the model, such as patient's eligibility for interventions, acceptability of interventions and availability of interventions, in order to ensure the interventions that they received were compatible with their attributes. To illustrate, for psychotic patients on antipsychotic medication, their history of antipsychotic medication use was recorded, including types of antipsychotic medication tried, duration of use, reasons for discontinuation, and whether the patients has developed certain adverse events while on the antipsychotic medication. This information was then compared against the algorithm for switching antipsychotic medication designed for the WDM to ensure any switch made sense given the data available.
- Check the behaviour of the entire model. For example, a unique numerical code was assigned to each workcentre in the model, allowing the complete pathway for individual patients to be recorded by saving the numerical codes of those workcentres that the patient passed through. The complete pathway was checked for the first 20 patients at CHR, the first 20 patients with psychosis and the first 20 patients who are not at risk.

A detailed record of all white box tests conducted is reported in eTable 8. Any errors or inconsistencies identified were checked and fixed.

© 2020 Jin H et al. *JAMA Network Open.*

eTable 8: White box tests conducted for the schizophrenia WDM

Types of model behaviour checked	Variables recorded	Records checked	Tests conducted
Behaviour of each workcentre			
**Attributes updated at each workcentre** (excluding costs and QALYs, which are reported separately below)	• Attributes at set up (e.g. patient's starting age, original disease status when entering the model, prognosis etc.)<br>• Attributes when entering the current workcentre (e.g. the last workcentre the patient passed through, patient's current age and disease status, remaining time to competing events, types and number of interventions received etc.)<br>• Attributes when leaving the current workcentre (e.g. the next workcentre the patient moves to, patient's current age and disease status after update, remaining time to events after update, types and number of interventions received after update etc.)<br>• Intermediate outcomes (e.g. temporary variables created to calculate how many antipsychotic medication depot injections the patient has received while staying in the current workcentre)	• The proportion of patients with CHR was set to 100%. The records for the first 1,000 patients were checked<br>• The proportion of patients with psychosis was set to 100%. The records for the first 1,000 patients were checked<br>• The proportion of patients not at risk of psychosis was set to 100%. The records for the first 1,000 patients were checked	The values of each attribute updated at the current workcentre were recalculated in EXCEL, and the calculated values were compared against the updated values produced by SIMUL8. For example:<br>• the next relevant event was calculated in EXCEL by comparing the remaining time to competing events when patient entering the current workcentre. The derived outcome was compared against the outcome produced by SIMUL8<br>• Based on the next relevant event and other relevant attributes, the patient's subsequent route was calculated in EXCEL and compared against the route generated by SIMUL8
**Costs (undiscounted and discounted)**	• Costs accrued during the current workcentre<br>• Total costs that the patient has accrued<br>• Patient's starting age when entering the model<br>• Patient's age when entering the current workcentre<br>• Patient's age when leaving the current workcentre	As above	Checked the plausibility of the undiscounted and discounted costs based on patients' disease status, prognosis, treatment plan and age when entering and leaving the workcentre
**LYGs and QALYs (undiscounted and discounted)**	• Patient's age when entering the current workcentre<br>• Patient's age when leaving the current workcentre<br>• Patient's disease status while staying in the current workcentre (which may be changed before patients leaving the current workcentre, e.g. patients at CHR who achieved recovery while receiving CBTs are assumed to continue therapy until they finish a full course)	As above	• The plausibility of the patient's age when entering and leaving the current workcentre were checked (e.g. patient's age when entering the current workcentre needs to be later than the patient's starting age and earlier than the patient's age

© 2020 Jin H et al. *JAMA Network Open.*

Types of model behaviour checked	Variables recorded	Records checked	Tests conducted
	• Patient's age when his/her disease status changes		when leaving the current workcentre)<br>• Patients LYGs and QALYs accrued whilst at the current workcentre were calculated by me and compared against the LYGs produced by SIMUL8<br>• The plausibility of the discounted LYGs and QALYs were checked and compared against the undiscounted LYGs and QALYs
Behaviour of multiple workcentres			
**Patient's treatment plan** (i.e. whether a patient will receive a specific intervention or not, including CBT, different types of antipsychotic medications and family interventions)	• Patient's eligibility for interventions<br>• Patient's acceptance of interventions<br>• Availability of interventions<br>• Patient's treatment plan produced by SIMUL8	• The proportion of patients with CHR was set to 100%. The records for the first 1,000 patients were checked<br>• The proportion of patients with psychosis was set to 100%. The records for the first 1,000 patients were checked<br>• The proportion of patients not at risk of psychosis was set to 100%. The records for the first 1,000 patients were checked	• The proportion of patients who are planned to receive a specific intervention were compared with patient's eligibility and acceptance of interventions, as well as availability of interventions<br>• Different scenarios were tested (e.g. 'Ideal scenario' and 'current practice' scenario) to check if the expected effect can be observed from the model outputs
**Interventions that patients actually received**	• Patient's treatment plan<br>• The actual type and number of interventions that the patient received	As above	Compared the type and number of interventions that the patient received with the original treatment plan
**Patient's pathway (i.e. the order of workcentres that a patient has passed)**	• Patient's starting and current disease status, and prognosis<br>• Patient's original treatment plan (determine based on patient's eligibility, acceptability of interventions and availability of interventions)<br>• A unique numerical code has been assigned to each workcentre in the model. Each patient's pathway was	The complete pathways were checked for:<br>• the first 20 patients entering the model as CHR<br>• the first 20 patients entering the model with psychosis	Checked the plausibility of a patient's pathway based on the patient's disease status, prognosis and treatment plan

© 2020 Jin H et al. *JAMA Network Open.*

Types of model behaviour checked	Variables recorded	Records checked	Tests conducted
	recorded by recoding the numerical codes of those workcentres that the patient has passed.	• the first 20 patients entering the model as not at risk of psychosis	
**Patient's use of antipsychotic medication and reason for discontinuation**	For each antipsychotic medication tried by the patient, the following information was recorded:<br>• Order of use (e.g. first-line or second-line)<br>• Time spent on a particular medication<br>• Reason for discontinuation (if applicable), including inefficacy, intolerability or non-adherence<br>• Whether the patient has developed adverse events while taking this antipsychotic medication, including weight gain, extrapyramidal symptoms (EPS), glucose intolerance, diabetes and neutropenia (if the patient is taking clozapine)	The records for the first 20 psychotic patients on antipsychotic medications were checked	Checked the use of antipsychotic medication and patient's response to antipsychotic medications
**Accumulated costs (undiscounted and discounted)**	• Type and number of interventions that the patient received<br>• Undiscounted and discounted cost accumulated at different parts of the pathway (e.g. cost accumulated while receiving family intervention, or while receiving acute treatment)	• The proportion of patients with CHR was set to 100%. The records for the first 1,000 patients were checked<br>• The proportion of patients with psychosis was set to 100%. The records for the first 1,000 patients were checked<br>• The proportion of patients not at risk of psychosis was set to 100%. The records for the first 1,000 patients were checked	• The plausibility of the undiscounted and discounted cost accumulated at different part of the pathway were checked based on the type and number of interventions that the patient received<br>• Different scenarios were tested (e.g. 'Ideal scenario' and 'current practice' scenario) to check if expected effect can be observed from the model outputs
**Accumulated LYGs and QALYs (undiscounted and discounted)**	• Patient's age when entering the model<br>• Patient's age of death<br>• Total accumulated LYGs and QALYs (undiscounted and discounted)<br>• LYGs and QALYs accumulated at different parts of the pathway	As above	• The difference between a patient's age of death and starting age was calculated in EXCEL and compared against the total accumulated LYGs produced by SIMUL8<br>• The plausibility of the LYGs and QALYs (undiscounted and

© 2020 Jin H et al. *JAMA Network Open.*

Types of model behaviour checked	Variables recorded	Records checked	Tests conducted
			discounted) accumulated at different parts of the pathway were checked<br>• Different scenarios were tested (e.g. 'Ideal scenario' and 'current practice' scenario) to check if the expected effect can be observed from the model outputs<br>• The values of all utilities were set to 1, to make sure QALYs=LYGs

© 2020 Jin H et al. *JAMA Network Open.*

5.2 Black-box tests conducted

Black-box tests were conducted to compare the baseline health and cost outputs of the schizophrenia WDM with published literature. The base case WDM ('current practice' scenario) was run for 100,000 simulated patients with possible psychosis. The following outputs were collected and reported below in the following order:

- Diagnostic outcomes (Section 5.2.1)
- Lifetime life-years gained (LYGs) and QALYs (Section 5.2.2)
- Lifetime costs (Section 5.2.3)

5.2.1 Diagnostic outcomes

The diagnostic outcomes when patients entered and left the model are reported in eTable 9, and graphically presented in eFigure 1. Patients enter the model when being referred to secondary care mental health services for possible psychosis and leave the model when they die. When patients first enter the model, about one third of them were assessed as CHR, one third were first-episode psychosis (FEP), and one third were not at risk of psychosis. These results are consistent with published UK literature [1]. When this cohort of patients left the model due to death, their diagnostic outcomes were recovered CHR (25.5%), recovered schizophrenia (6.4%), unrecovered schizophrenia (23.1%), non-schizophrenia psychosis (11.8%), and not at risk of psychosis (33.2%). These results are consistent with published literature [5,6].

© 2020 Jin H et al. *JAMA Network Open.*

eTable 9: Diagnostic outcomes when patients enter and leave the model

	**Number of patients (total n=100,000)** n (%)
**Diagnostic outcomes when patients enter the model**	
CHR	34,898 (34.9)
FEP	31,901 (31.9)
Not at risk of psychosis	33,201 (33.2)
**Diagnostic outcomes when patients left the model**	
Recovered CHR	25,499 (25.5)
Recovered schizophrenia	6,399 (6.4)
Unrecovered schizophrenia	23,100 (23.1)
Non-schizophrenia psychosis	11,801 (11.8)
Not at risk of psychosis	33,201 (33.2)

© 2020 Jin H et al. *JAMA Network Open.*

eFigure 1: Diagnostic outcomes when patients enter and leave the model

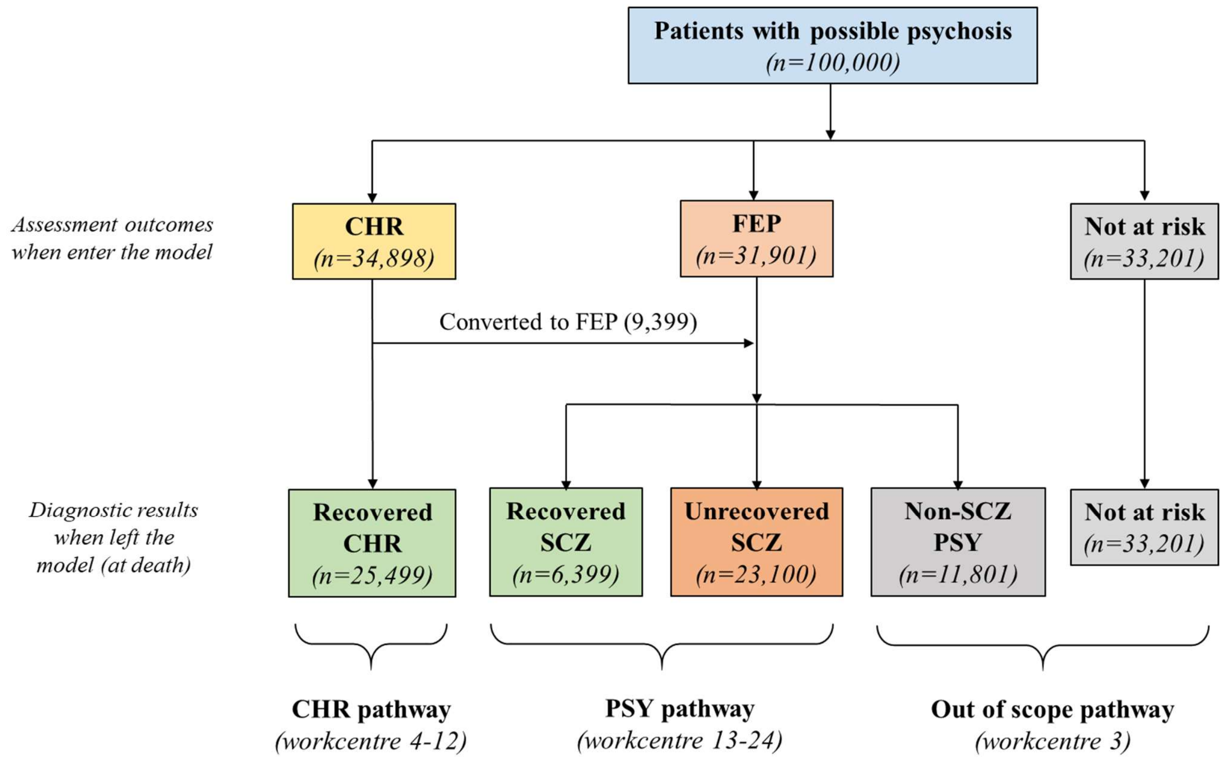

Abbreviations:
CHR: clinical high risk of psychosis; FEP: first episode of psychosis; Not at risk: not at risk of psychosis; SCZ: schizophrenia.

© 2020 Jin H et al. *JAMA Network Open.*

5.2.2 Lifetime LYGs and QALYs

The lifetime LYGs and QALYs for the same 100,000 simulated individuals are reported in eTable 10 and visually presented in eFigure 2. The results suggest that the largest contributor to overall LYGs relates to the out of scope pathway (47.4%), followed by the CHR pathway (30.1%), and the psychosis pathway (22.4%). The is due to the fact that of all patients referred for possible psychosis, the majority of them will not develop schizophrenia, including patients who are not at risk of psychosis (33.2%), recovered CHR (25.5%), and patients with non-schizophrenia psychosis (11.8%).

eTable 10: LYGs and QALYs estimated using the schizophrenia WDM[1]

	Undiscounted		Discounted	
	**LYGs (%)**	**QALYs (%)**	**LYGs (%)**	**QALYs (%)**
CHR pathway[2]	1,463,896 (30.1%)	1,248,917 (31.2%)	639,796 (28.1%)	561,011 (29.2%)
PSY pathway[3]	1,091,077 (22.4%)	759,505 (19.0%)	600,272 (26.3%)	424,417 (22.1%)
Out of scope[4]	2,305,721 (47.4%)	1,999,375 (49.9%)	1,040,001 (45.6%)	934,109 (48.7%)
**Total**	**4,860,693**	**4,007,797**	**2,280,069**	**1,919,536**

Abbreviations:
CHR: clinical high-risk of psychosis; LYG: life-year gained; PSY: psychosis; QALY: quality-adjusted life year.
Notes:
1. Results based on 100,000 simulated patients.
2. CHR pathway: workcentres 4-12.
3. PSY pathway: workcentres 13-24.
4. Out of scope pathway: workcentre 3.

eFigure 2: LYGs and QALYs estimated using the schizophrenia WDM[1]

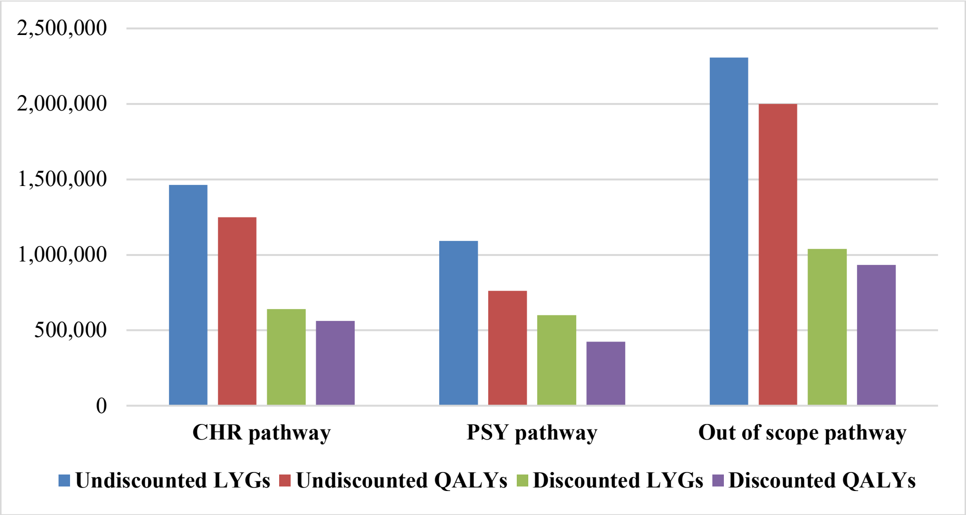

Abbreviations:
CHR: clinical high-risk of psychosis; LYG: life-year gained; PSY: psychosis; QALY: quality-adjusted life year.
Notes:
1. Results based on 100,000 simulated patients.

A break-down of the lifetime LYGs and QALYs by health states is reported in eTable 11. Within each part of the pathway, the order of magnitude of LYGs appear intuitively sensible, for example:

- Within the CHR pathway, the largest contributor comes from recovered CHR. This is consistent with previous findings that CHR is a transitional period which normally lasts less than 2 years and the majority of patients at CHR will not convert to psychosis [5,41].
- Within the psychosis pathway, for those patients who have not achieved clinical recovery, they spend about 10% of their time in the acute psychosis state (defined as a psychotic state which requires extensive care from psychiatric hospital or CRHT), and 90% of their time in the non-acute psychosis

© 2020 Jin H et al. *JAMA Network Open.*

state (defined as a psychotic state which does not require extensive care from psychiatric hospital or CRHT). Due to lack of data about the lifetime duration of time that psychotic patients spend in the acute and non-acute states, it is impossible to compare our findings with published studies. However, the stakeholders recruited for this PhD considered this finding to be intuitively sensible.

eTable 11: Breakdown of patients' LYGs by health states[1]

	**Undiscounted LYGs (%)**
CHR pathway *(workcentres 4-12)*	
Unconverted and unrecovered CHR	47,492 (3.2%)
Converted CHR	355 (0.0%)
Recovered CHR	1,416,049 (96.7%)
Total (CHR pathway)	**1,463,896**
PSY pathway *(workcentres 13-24)*	
Clinically recovered	213,892 (19.6%)
Unrecovered patients with acute psychosis	90,163 (8.3%)
Unrecovered patients with non-acute psychosis	787,022 (72.1%)
Total (PSY pathway)	**1,091,077**
Out of scope pathway *(workcentre 3)*	
Out of scope	2,305,721 (100.0%)
Total (out of scope pathway)	**2,305,721**

Notes:
1. Results based on 100,000 simulated patients.

5.2.3 Lifetime costs

eTable 12 presents lifetime costs for the same 100,000 simulated individuals by different parts of the pathway. The results suggest that the largest contributors to overall undiscounted cost are interventions for people with psychosis/ schizophrenia (98.8%), followed by end of life interventions (0.9%), mental health specialist assessments (0.1%) and the CHR pathway (0.1%). The reason why the cost of interventions for CHR is less than the cost of mental health specialist assessments and end of life interventions is likely to be that all patients in the model have used mental health specialist assessments (at least once) and end of life interventions, but only one third of those patients were assessed as CHR and thus eligible for interventions of CHR. And of those people at CHR, only 71.1% of them have access to and accept monitoring and only 20.9% have access to and accept CBT.

© 2020 Jin H et al. *JAMA Network Open.*

eTable 12: Lifetime costs estimated using the schizophrenia WDM[1]

	Undiscounted cost £ (%)	Discounted cost £ (%)
Mental health specialist assessments	38,217,286 (0.1%)	36,827,113 (0.2%)
Interventions for CHR[2]	24,696,960 (0.1%)	24,136,936 (0.1%)
Interventions for PSY[3]	26,855,848,666 (98.8%)	16,342,396,825 (99.3%)
End of life interventions	250,681,569 (0.9%)	54,059,664 (0.3%)
**Total**	**27,169,444,480**	**16,457,420,538**

Abbreviations:
CHR: clinical high-risk of psychosis; LYG: life-year gained; PSY: psychosis; QALY: quality-adjusted life year.
Notes:
1. Results based on 100,000 simulated patients.
2. Includes CBT and monitoring (if patients accept and have access to).
3. Includes antipsychotic medications, treatment of adverse events of antipsychotic medications, family interventions (if patients accept and have access to), interventions for assessing and managing acute psychosis (CRHT or hospital admission), outpatient appointments and residential care etc.

A break-down of the lifetime costs are reported in eTable 13 according to the broad groups of resources used within the service. Due to a lack of evidence about the lifetime cost of managing people at CHR or those people with a diagnosis of psychosis/schizophrenia, it is impossible to compare my results with published literature. However, the magnitude of costs within each part of the pathway appear to be intuitively sensible, according to the stakeholders recruited for this PhD. For example, within the psychosis pathway, the largest contributor to the total cost is treatment for acute psychosis (43.8%), followed by treatment for non-acute psychosis (42.8%). This is because although the daily cost of treating acute patients is much higher compared to the daily cost of treating stable patients, the average time that psychotic patients spend in acute status is only 10% of the time that patients spend in non-acute status.

© 2020 Jin H et al. *JAMA Network Open.*

eTable 13: Service costs estimated using the schizophrenia WDM[1]

	Undiscounted cost £ (%)	Discounted cost £ (%)
Assessment		
Mental health specialist assessment	38,217,286 (100.0%)	36,827,113 (100.0%)
Total (Assessment pathway)	**38,217,286**	**36,827,113**
Interventions for CHR		
CBT	10,776,943 (43.6%)	10,693,977 (44.3%)
Monitoring	13,920,018 (56.4%)	13,442,959 (55.7%)
Total (CHR pathway)	**24,696,960**	**24,136,936**
Interventions for people with psychosis/schizophrenia		
Treatment for acute psychosis[2]	11,910,879,446 (44.4%)	7,061,794,825 (43.2%)
Treatment for non-acute psychosis[3]	11,638,998,673 (43.3%)	6,378,209,184 (39.0%)
Treatment for recovered psychosis[3]	3,305,970,548 (12.3%)	2,902,392,817 (17.8%)
Total (Psychosis pathway)	**26,855,848,666**	**16,342,396,825**
End of life interventions		
End of life cost	250,681,569 (100.0%)	54,059,664 (100.0%)
**Total**	**27,169,444,475**	**16,457,420,535**

Notes:

1. Results based on 100,000 simulated patients.

2: Includes cost of assessing acute patients at CRHT, cost of CRHT or hospital care, antipsychotic medication (if patients accept), cost of treating adverse events of antipsychotic medication (weight gain, diabetes, EPS and neutropenia).

3: Includes cost of antipsychotic medication (if patients accept), treatment of adverse events (weight gain, diabetes, EPS and neutropenia), outpatient appointments (with psychiatrists, GPs, nurses, therapists or social workers), and residential care for those who need long-term care.

© 2020 Jin H et al. *JAMA Network Open.*

eAppendix 6. Results of Sensitivity Analysis

This section reports results of one-way, multi-way and structural sensitivity analysis for Topic A-E.

6.1 Results of sensitivity analysis for Topic A

eTable 14: Base case analysis, one-way and multi-way sensitivity analyses for Topic A

Intervention	Cost (£)	QALY	Incremental cost	Incremental QALY	ICER	Ranking of NMB (WTP=20,000 per QALY)	Ranking of NMB (WTP=30,000 per QALY)
Base case results (deterministic)							
PAU	168,695	19.1904	–	–	Dominated	2	2
PAU+CBT	167,452	19.1904	-1,243	0.0000	Dominating	1	1
SA 1: Assuming CBT can prevent transition to psychosis *(base case analysis assumes CBT can only delay transition to psychosis)*							
PAU	165,057	19.1950	–	–	Dominated	2	2
PAU+CBT	157,913	19.3593	-7,144	0.1643	Dominating	1	1
SA 2: Change RR of CBT = 0.63[1] *(baseline value:0.41)*							
PAU	168,695	19.1904	–	–	Dominated	2	2
PAU+CBT	168,194	19.1904	-501	0.0000	Dominating	1	1
SA 3: Set utility of people at CHR=0.9 *(baseline value:0.71)*							
PAU	168,695	19.2648	–	–	Dominated	2	2
PAU+CBT	167,452	19.2794	-1,243	0.0146	Dominating	1	1
SA 4: Brief CBT, assuming a reduced number of CBT sessions (8) and reduced effectiveness size, RR=0.71 *(base case analysis assumes a full course of CBT with 16 sessions and a RR of 0.41)*							
PAU	168,695	19.1904	–	–	Dominated	2	2
PAU+CBT	168,131	19.1904	-564	0.0000	Dominating	1	1
SA 5: Set unit cost of CBT = £139.83[2] *(baseline value: £97.00)*							
PAU	168,695	19.1904	–	–	Dominated	2	2
PAU+CBT	167,568	19.1904	-1,127	0.0000	Dominating	1	1

Abbreviations:
CBT: Cognitive behaviour therapy; ICER: incremental cost-effectiveness ratio; NMB: net monetary benefit; PAU: Practice-as-usual; QALY: quality-adjusted life of years; RR: relative risk; SA: sensitivity analysis; WTP=willingness-to-pay.

Notes:
1. 0.63 is the RR reported by the meta-analysis conducted by the NICE schizophrenia guideline [16].
2. The PSSRU reports a study which compares the unit cost of CBT reported by different studies [42]. For patients with psychosis, the highest unit cost reported is £105.62, which is equivalent to £139.83 in 2016/2017 value.

© 2020 Jin H et al. *JAMA Network Open.*

eTable 15: Structural sensitivity analyses for Topic A

Intervention	Cost (£)	QALY	Incremental cost	Incremental QALY	ICER	Ranking of NMB[1] (WTP=20,000 per QALY)	Ranking of NMB[1] (WTP=30,000 per QALY)
Configuration 1: *With all other topics set equal to the 'ideal scenario'*							
PAU	162,556	19.2101	–	–	Dominated	2	2
PAU+CBT	161,620	19.2103	-936	0.0002	Dominating	1	1
Configuration 2: *Topic B=100% hospital admission; Topic C=AP+FI (100% availability); Topic D=Placebo; Topic E=Clozapine with no delay*							
PAU	171,844	19.2070	–	–	Dominated	2	2
PAU+CBT	170,665	19.2074	-1,179	0.0004	Dominating	1	1
Configuration 3: *Topic B=100% hospital admission; Topic C=AP alone; Topic D=Amisulpride; Topic E=Clozapine with no delay*							
PAU	166,156	19.1793	–	–	Dominated	2	2
PAU+CBT	164,843	19.1806	-1,313	0.0013	Dominating	1	1
Configuration 4: *Topic B=100% hospital admission; Topic C=AP + FI (100% availability); Topic D=Amisulpride; Topic E=Clozapine with no delay*							
PAU	166,525	19.2000	–	–	Dominated	2	2
PAU+CBT	165,429	19.2001	-1,096	0.0001	Dominating	1	1
Configuration 5: *Topic B=100% hospital admission; Topic C=AP alone; Topic D=Placebo; Topic E=Clozapine with no delay*							
PAU	171,471	19.1984	–	–	Dominated	2	2
PAU+CBT	170,156	19.1988	-1,315	0.0004	Dominating	1	1
Configuration 6: *Topic B=100% hospital admission; Topic C=AP + FI (100% availability); Topic D=Placebo; Topic E=Clozapine with 3.98 year's delay*							
PAU	185,023	19.1933	–	–	Dominated	2	2
PAU+CBT	183,403	19.1947	-1,620	0.0014	Dominating	1	1
Configuration 7: *Topic B=100% hospital admission; Topic C=AP alone; Topic D=Amisulpride; Topic E=Clozapine with 3.98 year's delay*							
PAU	172,741	19.1733	–	–	Dominated	2	2
PAU+CBT	171,502	19.1747	-1,239	0.0014	Dominating	1	1
Configuration 8: *Topic B=100% hospital admission; Topic C=AP + FI (100% availability); Topic D=Amisulpride; Topic E=Clozapine with 3.98 year's delay*							
PAU	173,153	19.1937	–	–	Dominated	2	2
PAU+CBT	172,029	19.1941	-1,124	0.0004	Dominating	1	1
Configuration 9: *Topic B=100% hospital admission; Topic C=AP alone; Topic D=Placebo; Topic E=Clozapine with 3.98 year's delay*							
PAU	183,840	19.1845	–	–	Dominated	2	2
PAU+CBT	182,520	19.1861	-1,320	0.0016	Dominating	1	1

Abbreviations:
AP: antipsychotics; CBT: Cognitive behaviour therapy; ICER: Incremental cost-effectiveness ratio; NMB: net monetary benefit; PAU: Practice-as-usual; QALY: quality-adjusted life of years.

© 2020 Jin H et al. *JAMA Network Open.*

6.2 Results of sensitivity analysis for Topic B

eTable 16: Base case analysis, one-way and multi-way sensitivity analyses for Analysis Question 1 Topic B

Intervention	Cost (£)	QALY	Incremental cost	Incremental QALY	ICER	Ranking of NMB (WTP=20,000 per QALY)	Ranking of NMB (WTP=30,000 per QALY)
Base case results (deterministic)							
Mix of CRHT and hospital admission	168,078	19.1904	-3,655	0.000	Dominating	1	1
Hospital admission alone	171,733	19.1904	–	–	Dominated	2	2
SA 1: Set the portion of acute patients who can managed by CRHT=50% *(baseline value: ranges from 16.40% to 30.60%, depending on patients' age and sex)*							
Mix of CRHT and hospital admission	146,181	19.1904	-8,308	0.0000	Dominating	1	1
Hospital admission alone	154,489	19.1904	–	–	Dominated	2	2
SA 2: Set unit cost of a contact with the CRHT=£394.90 *(baseline value: £197.45)*							
Mix of CRHT and hospital admission	168,732	19.1904	-3,001	0.0000	Dominating	1	1
Hospital admission alone	171,733	19.1904	–	–	Dominated	2	2
SA 3: Set unit cost of hospital bed day=254[1] *(baseline value: £379.00)*							
Mix of CRHT and hospital admission	143,795	19.1904	-2,234	0.0000	Dominating	1	1
Hospital admission alone	146,029	19.1904	–	–	Dominated	2	2
SA 4: Set the mean duration of hospital stays=90.73 days[2] *(baseline value: 138.90 days)*							
Mix of CRHT and hospital admission	157,378	19.2016	-2,157	0.0000	Dominating	1	1
Hospital admission alone	159,535	19.2016	–	–	Dominated	2	2

Notes:

1. NHS reference cost 2015-2016: code WD22Z 'All patients between 19 and 69 years with a Mental Health Primary Diagnosis, treated by a Non-Specialist Mental Health Service Provider' [35].
2. Calculated from data reported in UK trial [43].

Abbreviations:

CRHT: Crisis resolution and home treatment team. ICER: incremental cost-effectiveness ratio; NMB: net monetary benefit; WTP: willingness-to-pay.

© 2020 Jin H et al. *JAMA Network Open.*

eTable 17: Structural sensitivity analyses for Analysis Question 1 Topic B

Intervention	Cost (£)	QALY	Incremental cost	Incremental QALY	ICER	Ranking of NMB (WTP=20,000 per QALY)	Ranking of NMB (WTP=30,000 per QALY)
With all other topics set equal to the 'ideal scenario'							
Mix of CRHT and hospital admission	161,620	19.2103	-3,135	0.0000	Dominating	1	1
Hospital admission alone	164,755	19.2103	–	–	Dominated	2	2
Configuration 1: *Topic A=CBT (100% availability); Topic C=AP + FI (100% availability); Topic D=Placebo; Topic E=Clozapine with no delay*							
Mix of CRHT and hospital admission	167,296	19.2074	-3,369	0.0000	Dominating	1	1
Hospital admission alone	170,665	19.2074	–	–	Dominated	2	2
Configuration 2: *Topic A=CBT (100% availability); Topic C=AP alone; Topic D=Amisulpride; Topic E=Clozapine with no delay*							
Mix of CRHT and hospital admission	161,630	19.1806	-3,213	0.0000	Dominating	1	1
Hospital admission alone	164,843	19.1806	–	–	Dominated	2	2
Configuration 3: *Topic A=CBT (100% availability); Topic C=AP + FI (100% availability); Topic D=Amisulpride; Topic E=Clozapine with no delay*							
Mix of CRHT and hospital admission	162,218	19.2001	-3,211	0.0000	Dominating	1	1
Hospital admission alone	165,429	19.2001	–	–	Dominated	2	2
Configuration 4: *Topic A=CBT (100% availability); Topic C=AP alone; Topic D=Placebo; Topic E=Clozapine with no delay*							
Mix of CRHT and hospital admission	166,780	19.1988	-3,376	0.0000	Dominating	1	1
Hospital admission alone	170,156	19.1988	–	–	Dominated	2	2
Configuration 5: *Topic A=CBT (100% availability); Topic C=AP + FI (100% availability); Topic D=Placebo; Topic E=Clozapine with 3.98 year's delay*							
Mix of CRHT and hospital admission	179,201	19.1947	-4,202	0.0000	Dominating	1	1
Hospital admission alone	183,403	19.1947	–	–	Dominated	2	2
Configuration 6: *Topic A=CBT (100% availability); Topic C=AP alone; Topic D=Placebo; Topic E=Clozapine with 3.98 year's delay*							
Mix of CRHT and hospital admission	167,831	19.1747	-3,671	0.0000	Dominating	1	1
Hospital admission alone	171,502	19.1747	–	–	Dominated	2	2
Configuration 7: *Topic A=CBT (100% availability); Topic C=AP + FI (100% availability); Topic D=Amisulpride; Topic E=Clozapine with 3.98 year's delay*							
Mix of CRHT and hospital admission	168,353	19.1941	-3,676	0.0000	Dominating	1	1
Hospital admission alone	172,029	19.1941	–	–	Dominated	2	2
Configuration 8: *Topic A=CBT (100% availability); Topic C=AP alone; Topic D=Placebo; Topic E=Clozapine with 3.98 year's delay*							
Mix of CRHT and hospital admission	178,308	19.1861	-4,212	0.0000	Dominating	1	1
Hospital admission alone	182,520	19.1861	–	–	Dominated	2	2
Configuration 9: *Topic A=No CBT; Topic C=AP + FI (100% availability); Topic D=Placebo; Topic E=Clozapine with no delay*							
Mix of CRHT and hospital admission	168,457	19.2070	-3,387	0.0000	Dominating	1	1
Hospital admission alone	171,844	19.2070	–	–	Dominated	2	2
Configuration 10: *Topic A=No CBT; Topic C=AP alone; Topic D=Amisulpride; Topic E=Clozapine with no delay*							
Mix of CRHT and hospital admission	162,919	19.1793	-3,237	0.0000	Dominating	1	1
Hospital admission alone	166,156	19.1793	–	–	Dominated	2	2
Configuration 11: *Topic A=No CBT; Topic C=AP + FI (100% availability); Topic D=Amisulpride; Topic E=Clozapine with no delay*							
Mix of CRHT and hospital admission	163,302	19.2000	-3,223	0.0000	Dominating	1	1
Hospital admission alone	166,525	19.2000	–	–	Dominated	2	2

© 2020 Jin H et al. *JAMA Network Open.*

Intervention	Cost (£)	QALY	Incremental cost	Incremental QALY	ICER	Ranking of NMB (WTP=20,000 per QALY)	Ranking of NMB (WTP=30,000 per QALY)
Configuration 12: *Topic A=No CBT; Topic C=AP alone; Topic D=Placebo; Topic E=Clozapine with no delay*							
Mix of CRHT and hospital admission	168,097	19.1984	-3,374	0.0000	Dominating	1	1
Hospital admission alone	171,471	19.1984	–	–	Dominated	2	2
Configuration 13: *Topic A=No CBT; Topic C=AP + FI (100% availability); Topic D=Placebo; Topic E=Clozapine with 3.98 year's delay*							
Mix of CRHT and hospital admission	180,781	19.1933	-4,242	0.0000	Dominating	1	1
Hospital admission alone	185,023	19.1933	–	–	Dominated	2	2
Configuration 14: *Topic A=No CBT; Topic C=AP alone; Topic D=Amisulpride; Topic E=Clozapine with 3.98 year's delay*							
Mix of CRHT and hospital admission	169,045	19.1733	-3,696	0.0000	Dominating	1	1
Hospital admission alone	172,741	19.1733	–	–	Dominated	2	2
Configuration 15: *Topic A=No CBT; Topic C=AP + FI (100% availability); Topic D=Amisulpride; Topic E=Clozapine with 3.98 year's delay*							
Mix of CRHT and hospital admission	169,470	19.1937	-3,683	0.0000	Dominating	1	1
Hospital admission alone	173,153	19.1937	–	–	Dominated	2	2
Configuration 16: *Topic A=No CBT; Topic C=AP alone; Topic D=Placebo; Topic E=Clozapine with 3.98 year's delay*							
Mix of CRHT and hospital admission	179,603	19.1845	-4,237	0.0000	Dominating	1	1
Hospital admission alone	183,840	19.1845	–	–	Dominated	2	2

Abbreviations:
AP: antipsychotic medication; FI: family intervention; ICER: incremental cost-effectiveness ratio; WTP: willingness-to-pay.

© 2020 Jin H et al. *JAMA Network Open.*

6.3 Results of sensitivity analysis for Topic C

eTable 18: Base case analysis, one-way and multi-way sensitivity analyses for Analysis Question 1 Topic C

Intervention	Cost (£)	QALY	Incremental cost	Incremental QALY	ICER	Ranking of NMB (WTP=20,000 per QALY)	Ranking of NMB (WTP=30,000 per QALY)
Base case results (deterministic)							
Olanzapine	167,455	19.1794	–	–	Dominated	3	3
Amisulpride	165,813	19.1822	–	–	–	1	1
Placebo	174,128	19.1931	–	–	Dominated	7	7
Risperidone	166,869	19.1934	1,056	0.0112	94,286	2	2
Aripiprazole	171,340	19.1977	–	–	Dominated	6	6
Haloperidol	168,538	19.1981	–	–	Extendedly dominated	5	5
Quetiapine	168,539	19.2005	1,670	0.0071	235,211	4	4
SA 1: Set the daily cost of all antipsychotic medications=£1 *(Baseline value: Amisulpride=£0.47, Aripiprazole=£4.08, Haloperidol=£0.37, Olanzapine=£0.13, Quetiapine=£1.24, Risperidone=£0.36)*							
Olanzapine	166,649	19.1794	–	–	Dominated	3	3
Amisulpride	164,730	19.1822	–	–	–	1	1
Placebo	173,378	19.1931	–	–	Dominated	7	7
Risperidone	165,989	19.1934	1,259	0.0112	112,995	2	2
Aripiprazole	168,059	19.1977	–	–	Dominated	6	6
Haloperidol	167,630	19.1981	–	–	Dominated	5	5
Quetiapine	167,239	19.2005	1,250	0.0071	174,953	4	4
SA 2: Exclude the cost and health impacts of all adverse events *(including weight gain, EPS, glucose intolerance and diabetes)*							
Olanzapine	166,969	19.2463	–	–	Dominated	3	3
Amisulpride	165,129	19.2481	–	–	Dominating	1	1
Placebo	173,788	19.2387	–	–	Dominated	7	7
Risperidone	166,383	19.2471	–	–	Dominated	2	2
Aripiprazole	170,924	19.2448	–	–	Dominated	6	6
Haloperidol	168,116	19.2452	–	–	Dominated	4	4
Quetiapine	168,171	19.2459	–	–	Dominated	5	5
SA 3: Exclude the cost and health impacts of weight gain							
Olanzapine	167,283	19.2266	519	0.0041	126,585	3	3
Amisulpride	165,710	19.2106	–	–	–	1	1
Placebo	174,028	19.2227	–	–	Dominated	7	7
Risperidone	166,764	19.2225	1,054	0.0119	88,571	2	2
Aripiprazole	171,247	19.2236	–	–	Dominated	6	6
Haloperidol	168,469	19.2179	–	–	Dominated	5	5
Quetiapine	168,439	19.2286	1,156	0.002	578,000	4	4
SA 4: Exclude the cost and health impacts of EPS							
Olanzapine	167,450	19.185	–	–	Dominated	3	3

© 2020 Jin H et al. *JAMA Network Open.*

Intervention	Cost (£)	QALY	Incremental cost	Incremental QALY	ICER	Ranking of NMB (WTP=20,000 per QALY)	Ranking of NMB (WTP=30,000 per QALY)
Amisulpride	165,806	19.1906	–	–	–	1	1
Placebo	174,123	19.2002	–	–	Dominated	7	7
Risperidone	166,863	19.2004	1,057	0.0098	107,857	2	2
Aripiprazole	171,335	19.2045	–	–	Dominated	6	6
Haloperidol	168,530	19.2093	1,667	0.0089	187,303	4	4
Quetiapine	168,535	19.2064	–	–	Dominated	5	5
SA 5: Exclude the cost and health impacts of glucose intolerance and diabetes							
Olanzapine	167,192	19.1936	–	–	Dominated	3	3
Amisulpride	165,265	19.2113	–	–	–	1	1
Placebo	173,949	19.2021	–	–	Dominated	7	7
Risperidone	166,537	19.211	–	–	Dominated	2	2
Aripiprazole	171,066	19.2122	–	–	Dominated	6	6
Haloperidol	168,236	19.2143	2,971	0.003	990,333	4	4
Quetiapine	168,322	19.212	–	–	Dominated	5	5

Abbreviations:
ICER: incremental cost-effectiveness ratio; NMB: net monetary benefit; WTP: willingness-to-pay.

© 2020 Jin H et al. *JAMA Network Open.*

eTable 19: Structural sensitivity analyses for Analysis Question 1 Topic C

Intervention	Cost (£)	QALY	Incremental cost	Incremental QALY	ICER	Ranking of NMB (WTP=20,000 per QALY)	Ranking of NMB (WTP=30,000 per QALY)
Configuration 1: *With all other topics set equal to the 'ideal scenario'*							
Olanzapine	160,135	19.2032	–	–	–	1	1
Amisulpride	162,218	19.2001	–	–	Dominated	4	4
Placebo	167,296	19.2074	–	–	Dominated	7	7
Risperidone	160,642	19.2115	507	0.0083	61,084	2	2
Aripiprazole	165,065	19.2146	–	–	Dominated	6	6
Haloperidol	162,775	19.2136	–	–	Dominated	5	5
Quetiapine	162,297	19.2176	1,655	0.0061	271,311	3	3
Configuration 2: *Topic A=CBT (100% availability); Topic B=100% hospital admission; Topic D=AP+FI (100% availability); Topic E=Clozapine with no delay*							
Olanzapine	163,239	19.2032	–	–	–	1	1
Amisulpride	165,429	19.2001	–	–	Dominated	4	4
Placebo	170,665	19.2074	–	–	Dominated	7	7
Risperidone	163,790	19.2115	551	0.0083	66,386	2	2
Aripiprazole	168,270	19.2146	–	–	Dominated	6	6
Haloperidol	165,972	19.2136	–	–	Dominated	5	5
Quetiapine	165,469	19.2176	1,679	0.0061	275,246	3	3
Configuration 3: *Topic A=CBT (100% availability); Topic B=100% hospital admission; Topic D=AP alone; Topic E=Clozapine with no delay*							
Olanzapine	163,405	19.1804	–	–	–	1	1
Amisulpride	164,843	19.1806	–	–	Dominated	3	4
Placebo	170,156	19.1988	–	–	Dominated	7	7
Risperidone	163,931	19.1953	526	0.0149	35,302	2	2
Aripiprazole	167,918	19.2003	–	–	Dominated	6	6
Haloperidol	165,477	19.2012	–	–	Dominated	5	5
Quetiapine	165,401	19.2039	1,470	0.0086	170,930	4	3
Configuration 4: *Topic A=No CBT; Topic B=100% hospital admission; Topic D=AP+FI (100% availability); Topic E=Clozapine with no delay*							
Olanzapine	164,136	19.2031	–	–	–	1	1
Amisulpride	166,525	19.2	–	–	Dominated	4	4
Placebo	171,844	19.207	–	–	Dominated	7	7
Risperidone	164,830	19.2113	694	0.0082	84,634	2	2
Aripiprazole	169,297	19.2144	–	–	Dominated	6	6
Haloperidol	166,980	19.2132	–	–	Dominated	5	5
Quetiapine	166,490	19.2172	1,660	0.0059	281,356	3	3
Configuration 5: *Topic A=No CBT; Topic B=100% hospital admission; Topic D=AP alone; Topic E=Clozapine with no delay*							
Olanzapine	164,459	19.18	–	–	–	1	1
Amisulpride	166,156	19.1793	–	–	Dominated	3	5
Placebo	171,471	19.1984	–	–	Dominated	7	7

© 2020 Jin H et al. *JAMA Network Open.*

Intervention	Cost (£)	QALY	Incremental cost	Incremental QALY	ICER	Ranking of NMB (WTP=20,000 per QALY)	Ranking of NMB (WTP=30,000 per QALY)
Risperidone	164,997	19.1946	–	–	Extendedly dominated	2	2
Aripiprazole	169,069	19.1997	–	–	Dominated	6	6
Haloperidol	166,676	19.2011	–	–	Extendedly dominated	5	4
Quetiapine	166,681	19.2025	2,222	0.0225	98,756	4	3
Configuration 6: *Topic A=CBT (100% availability); Topic B=100% hospital admission; Topic D=AP+FI (100% availability); Topic E=Clozapine with 3.98 year's delay*							
Olanzapine	173,540	19.1933	–	–	Dominated	2	2
Amisulpride	172,029	19.1941	–	–	–	1	1
Placebo	183,403	19.1947	–	–	Dominated	7	7
Risperidone	173,905	19.2019	1,876	0.0078	240,513	3	3
Aripiprazole	179,050	19.2038	–	–	Dominated	6	6
Haloperidol	175,912	19.2036	2,007	0.0017	1,180,588	4	4
Quetiapine	178,478	19.2045	2,566	0.0009	2,851,111	5	5
Configuration 7: *Topic A=CBT (100% availability); Topic B=100% hospital admission; Topic D=AP alone; Topic E=Clozapine with 3.98 year's delay*							
Olanzapine	173,486	19.1707	–	–	Dominated	3	3
Amisulpride	171,502	19.1747	–	–	–	1	1
Placebo	182,520	19.1861	–	–	Dominated	7	7
Risperidone	173,662	19.1849	2,160	0.0102	211,765	2	2
Aripiprazole	178,389	19.1895	–	–	Dominated	6	6
Haloperidol	175,420	19.1904	1,758	0.0055	319,636	4	4
Quetiapine	177,967	19.1903	–	–	Dominated	5	5
Configuration 8: *Topic A=No CBT; Topic B=100% hospital admission; Topic D=AP+FI (100% availability); Topic E=Clozapine with 3.98 year's delay*							
Olanzapine	174,763	19.1923	–	–	Dominated	2	2
Amisulpride	173,153	19.1937	–	–	–	1	1
Placebo	185,023	19.1933	–	–	Dominated	7	7
Risperidone	175,214	19.2021	2,061	0.0084	245,357	3	3
Aripiprazole	180,261	19.2033	–	–	Dominated	6	6
Haloperidol	176,933	19.2031	1,719	0.001	1,719,000	4	4
Quetiapine	179,342	19.2041	2,409	0.001	2,409,000	5	5
Configuration 9: *Topic A=No CBT; Topic B=100% hospital admission; Topic D=AP alone; Topic E=Clozapine with 3.98 year's delay*							
Olanzapine	174,822	19.169	–	–	Dominated	3	3
Amisulpride	172,741	19.1733	–	–	–	1	1
Placebo	183,840	19.1845	–	–	Dominated	7	7
Risperidone	174,982	19.1845	2,241	0.0112	200,089	2	2
Aripiprazole	179,499	19.189	–	–	Dominated	6	6
Haloperidol	176,559	19.1912	1,577	0.0067	235,373	4	4
Quetiapine	178,882	19.1899	–	–	Dominated	5	5

© 2020 Jin H et al. *JAMA Network Open.*

Abbreviations:
AP: antipsychotic medication; FI: family intervention; ICER: incremental cost-effectiveness ratio; NMB: net monetary benefit; WTP: willingness-to-pay.

© 2020 Jin H et al. *JAMA Network Open.*

6.4 Results of sensitivity analysis for Topic D

eTable 20: Base case analysis, one-way and multi-way sensitivity analyses for Topic D

Intervention	Cost (£)	QALY	Incremental cost	Incremental QALY	ICER	Ranking of NMB (WTP=20,000 per QALY)	Ranking of NMB (WTP=30,000 per QALY)
Base case results (deterministic)							
Antipsychotic medication alone	168,261	19.1849	–	–	Dominated	2	2
Family intervention alone	175,065	19.1987	–	–	Dominated	3	3
Antipsychotic medication + family intervention	167,905	19.2033	–	–	Dominating	1	1
SA 1: Exclude the cost and health impacts of all adverse events of antipsychotic medication *(including weight gain, EPS, glucose intolerance and diabetes)*							
Antipsychotic medication alone	167,776	19.2457	–	–	Dominated	2	2
Family intervention alone	174,762	19.2390	–	–	Dominated	3	3
Antipsychotic medication + family intervention	167,551	19.2476	–	–	Dominating	1	1
SA 2: Set RR of family intervention=0.83[1] *(Baseline value:0.63)*							
Antipsychotic medication alone	168,261	19.1849	–	–	–	1	1
Family intervention alone	175,741	19.1969	–	–	Dominated	3	3
Antipsychotic medication + family intervention	169,458	19.2013	1,197	0.0164	72,988	2	2
SA 3: Brief family intervention, assuming a shortened course with reduced number of sessions (10) and reduced effectiveness size (RR=0.82)<br>*(Base case analysis assumes a full course of family intervention with 20 sessions and a RR of 0.63)*							
Antipsychotic medication alone	168,261	19.1849	–	–	–	1	1
Family intervention alone	176,133	19.1895	–	–	Dominated	3	3
Antipsychotic medication + family intervention	169,373	19.1916	1,112	0.0067	165,970	2	2
SA 4: Set unit cost of family intervention=£224.00 *(Baseline value: £112.00)*							
Antipsychotic medication alone	168,261	19.1849	–	–	Dominated	2	2
Family intervention alone	175,333	19.1987	–	–	Dominated	3	3
Antipsychotic medication + family intervention	168,175	19.2033	–	–	Dominating	1	1

Abbreviations:
ICER: incremental cost-effectiveness ratio; NMB: net monetary benefit; QALY: quality-adjusted life of years; RR: relative risk; SA: sensitivity analysis; WTP=willingness-to-pay.
Notes:
1. This is the 2-5 year RR reported by the systematic review conducted by the NICE schizophrenia GDG [16].

© 2020 Jin H et al. *JAMA Network Open.*

eTable 21: Structural sensitivity analyses for Topic D

Intervention	Cost (£)	QALY	Incremental cost	Incremental QALY	ICER	Ranking of NMB[1] (WTP=20,000 per QALY)	Ranking of NMB[1] (WTP=30,000 per QALY)
Configuration 1: *With all other topics set equal to the 'ideal scenario'*							
Antipsychotic medication alone	161,418	19.1930	–	–	–	2	2
Family intervention alone	167,296	19.2074	–	–	Dominated	3	3
Antipsychotic medication + family intervention	161,620	19.2103	202	0.0173	11,676	1	1
Configuration 2: *Topic A=CBT (100% availability); Topic B=100% hospital admission; Topic D=Amisulpride; Topic E=Clozapine with no delay*							
Antipsychotic medication alone	164,843	19.1806	–	–	–	1	1
Family intervention alone	170,665	19.2074	5,236	0.0073	717,260	3	3
Antipsychotic medication + family intervention	165,429	19.2001	586	0.0195	30,051	2	2
Configuration 3: *Topic A=No CBT; Topic B=100% hospital admission; Topic D=Amisulpride; Topic E=Clozapine with 3.98 year's delay*							
Antipsychotic medication alone	172,741	19.1733	–	–	–	1	2
Family intervention alone	185,023	19.1933	–	–	Dominated	3	3
Antipsychotic medication + family intervention	173,153	19.1937	412	0.0204	20,196	2	1
Configuration 4: *Topic A=CBT (100% availability); Topic B=100% hospital admission; Topic D=Amisulpride; Topic E=Clozapine with 3.98 year's delay*							
Antipsychotic medication alone	171,502	19.1747	–	–	–	1	2
Family intervention alone	183,403	19.1947	11,374	0.0006	18,956,667	3	3
Antipsychotic medication + family intervention	172,029	19.1941	527	0.0194	27,165	2	1
Configuration 5: *Topic A=No CBT; Topic B=100% hospital admission; Topic D=Amisulpride; Topic E=Clozapine with no delay*							
Antipsychotic medication alone	166,156	19.1793	–	–	–	2	2
Family intervention alone	171,844	19.2070	5,319	0.007	759,857	3	3
Antipsychotic medication + family intervention	166,525	19.2000	369	0.0207	17,826	1	1

Abbreviations:
CBT: Cognitive behaviour therapy; ICER: incremental cost-effectiveness ratio; NMB: net monetary benefit; QALY: quality-adjusted life of years; WTP=willingness-to-pay.

© 2020 Jin H et al. *JAMA Network Open.*

6.5 Results of sensitivity analysis for Topic E

eTable 22: Base case analysis, one-way and multi-way sensitivity analyses for Topic E

Intervention	Cost (£)	QALY	Incremental cost	Incremental QALY	ICER	Ranking of NMB (WTP=20,000 per QALY)	Ranking of NMB (WTP=30,000 per QALY)
Base case results (deterministic)							
Quetiapine	172,043	19.1867	–	–	Dominated	5	5
Haloperidol	170,008	19.1883	–	–	Dominated	4	4
Risperidone	169,324	19.1889	–	–	Dominated	3	3
Olanzapine	165,444	19.1925	–	–	Dominated	2	2
Clozapine	162,215	19.1977	–	–	Dominating	1	1
SA 1: Set the daily cost of all antipsychotic medication=£1<br>*(Baseline value: Clozapine=£1.56, Olanzapine=£0.13, Risperidone=£0.36, Haloperidol=£0.37, Quetiapine=£1.24)*							
Quetiapine	170,642	19.1867	–	–	Dominated	5	5
Haloperidol	168,736	19.1883	–	–	Dominated	4	4
Risperidone	168,109	19.1889	–	–	Dominated	3	3
Olanzapine	164,312	19.1925	–	–	Dominated	2	2
Clozapine	160,748	19.1977	–	–	Dominating	1	1
SA 2: Assuming clozapine can reduce all-cause mortality with a hazard ratio=0.35 *(Base case analysis assumes clozapine has not impact on all-cause mortality)*							
Quetiapine	181,732	19.4882	–	–	Dominated	5	5
Haloperidol	180,472	19.518	–	–	Dominated	4	4
Risperidone	176,475	19.4125	–	–	Extendedly dominated	3	3
Olanzapine	170,775	19.3593			–	1	2
Clozapine	179,989	19.7607	9,214	0.4014	22,955	2	1
SA 3: Exclude the cost and health impacts of all adverse events *(including weight gain, EPS, glucose intolerance, diabetes and neutropenia)*							
Quetiapine	171,597	19.2422	–	–	Dominated	5	5
Haloperidol	169,558	19.2443	–	–	Dominated	4	4
Risperidone	168,892	19.2448	–	–	Dominated	3	3
Olanzapine	165,016	19.2488	–	–	Dominated	2	2
Clozapine	161,676	19.2529	–	–	Dominating	1	1
SA 4: Exclude the cost and health impacts of weight gain							
Quetiapine	171,918	19.2215	–	–	Dominated	5	5
Haloperidol	169,881	19.2235	–	–	Dominated	4	4
Risperidone	169,197	19.224	–	–	Dominated	3	3
Olanzapine	165,314	19.2283	–	–	Dominated	2	2
Clozapine	162,086	19.2329	–	–	Dominating	1	1
SA 5: Exclude the cost and health impacts of EPS							
Quetiapine	172,039	19.1927	–	–	Dominated	5	5
Haloperidol	170,003	19.1949	–	–	Dominated	4	4
Risperidone	169,319	19.1951	–	–	Dominated	3	3

© 2020 Jin H et al. *JAMA Network Open.*

Intervention	Cost (£)	QALY	Incremental cost	Incremental QALY	ICER	Ranking of NMB (WTP=20,000 per QALY)	Ranking of NMB (WTP=30,000 per QALY)
Olanzapine	165,440	19.1985	–	–	Dominated	2	2
Clozapine	162,210	19.2034	–	–	Dominating	1	1
SA 6: Exclude the cost and health impacts of glucose intolerance and diabetes							
Quetiapine	171,770	19.1867	–	–	Dominated	5	5
Haloperidol	169,739	19.1883	–	–	Dominated	4	4
Risperidone	169,054	19.1889	–	–	Dominated	3	3
Olanzapine	165,172	19.1925	–	–	Dominated	2	2
Clozapine	161,945	19.1977	–	–	Dominating	1	1
SA 7: Exclude the cost and health impacts of neutropenia							
Quetiapine	171,999	19.1867	–	–	Dominated	5	5
Haloperidol	169,959	19.1883	–	–	Dominated	4	4
Risperidone	169,293	19.1889	–	–	Dominated	3	3
Olanzapine	165,422	19.1925	–	–	Dominated	2	2
Clozapine	162,078	19.1977	–	–	Dominating	1	1

Abbreviations:
ICER: incremental cost-effectiveness ratio; NMB: net monetary benefit; QALY: quality-adjusted life of years; SA: sensitivity analysis; WTP=willingness-to-pay.

© 2020 Jin H et al. *JAMA Network Open.*

eTable 23: Structural sensitivity analyses for Topic E

Intervention	Cost (£)	QALY	Incremental cost	Incremental QALY	ICER	Ranking of NMB (WTP=20,000 per QALY)	Ranking of NMB (WTP=30,000 per QALY)
With all other topics set equal to the 'ideal scenario'							
Quetiapine	171,802	19.1992	–	–	Dominated	5	5
Haloperidol	168,899	19.2012	–	–	Dominated	4	4
Risperidone	168,783	19.2019	–	–	Dominated	3	3
Olanzapine	164,750	19.2056	–	–	Dominated	2	2
Clozapine	161,620	19.2103	–	–	Dominating	1	1
Configuration 1: *Topic A=CBT (100% availability); Topic B=100% hospital admission; Topic C=AP+FI (100% availability); Topic D=Placebo*							
Quetiapine	183,403	19.1947	–	–	Dominated	5	5
Haloperidol	181,015	19.196	–	–	Dominated	4	4
Risperidone	180,275	19.1967	–	–	Dominated	3	3
Olanzapine	174,873	19.2009	–	–	Dominated	2	2
Clozapine	170,665	19.2074	–	–	Dominating	1	1
Configuration 2: *Topic A=CBT (100% availability); Topic B=100% hospital admission; Topic C=AP alone; Topic D=Placebo*							
Quetiapine	182,520	19.1861	–	–	Dominated	5	5
Haloperidol	179,934	19.1876	–	–	Dominated	4	4
Risperidone	179,003	19.1884	–	–	Dominated	3	3
Olanzapine	173,868	19.1922	–	–	Dominated	2	2
Clozapine	170,156	19.1988	–	–	Dominating	1	1
Configuration 3: *Topic A=No CBT; Topic B=100% hospital admission; Topic C=AP+FI (100% availability); Topic D=Placebo*							
Quetiapine	185,023	19.1933	–	–	Dominated	5	5
Haloperidol	182,055	19.1955	–	–	Dominated	4	4
Risperidone	181,272	19.1964	–	–	Dominated	3	3
Olanzapine	175,965	19.2009	–	–	Dominated	2	2
Clozapine	171,844	19.207	–	–	Dominating	1	1
Configuration 4: *Topic A=No CBT; Topic B=100% hospital admission; Topic C=AP alone; Topic D=Placebo*							
Quetiapine	183,840	19.1845	–	–	Dominated	5	5
Haloperidol	180,868	19.1878	–	–	Dominated	4	4
Risperidone	180,080	19.1876	–	–	Dominated	3	3
Olanzapine	175,225	19.192	–	–	Dominated	2	2
Clozapine	171,471	19.1984	–	–	Dominating	1	1
Configuration 5: *Topic A=CBT (100% availability); Topic B=100% hospital admission; Topic C=AP+FI (100% availability); Topic D=Amisulpride*							
Quetiapine	172,029	19.1941	–	–	Dominated	5	5
Haloperidol	170,432	19.1954	–	–	Dominated	4	4
Risperidone	169,993	19.1952	–	–	Dominated	3	3
Olanzapine	167,673	19.1974	–	–	Dominated	2	2
Clozapine	165,429	19.2001	–	–	Dominating	1	1

© 2020 Jin H et al. *JAMA Network Open.*

Intervention	Cost (£)	QALY	Incremental cost	Incremental QALY	ICER	Ranking of NMB (WTP=20,000 per QALY)	Ranking of NMB (WTP=30,000 per QALY)
***Configuration 6:** Topic A=CBT (100% availability); Topic B=100% hospital admission; Topic C=AP alone; Topic D=Amisulpride*							
Quetiapine	171,502	19.1747	–	–	Dominated	5	5
Haloperidol	170,086	19.1752	–	–	Dominated	4	4
Risperidone	169,469	19.1761	–	–	Dominated	3	3
Olanzapine	167,084	19.1777	–	–	Dominated	2	2
Clozapine	164,843	19.1806	–	–	Dominating	1	1
***Configuration 7:** Topic A=No CBT; Topic B=100% hospital admission; Topic C=AP+FI (100% availability); Topic D=Amisulpride*							
Quetiapine	173,153	19.1937	–	–	Dominated	5	5
Haloperidol	171,477	19.1949	–	–	Dominated	4	4
Risperidone	171,205	19.1949	–	–	Dominated	3	3
Olanzapine	168,754	19.1963	–	–	Dominated	2	2
Clozapine	166,525	19.2000	–	–	Dominating	1	1
***Configuration 8:** Topic A=No CBT; Topic B=100% hospital admission; Topic C=AP alone; Topic D=Amisulpride*							
Quetiapine	172,741	19.1733	–	–	Dominated	5	5
Haloperidol	171,136	19.1743	–	–	Dominated	4	4
Risperidone	170,788	19.1749	–	–	Dominated	3	3
Olanzapine	168,165	19.1772	–	–	Dominated	2	2
Clozapine	166,156	19.1793	–	–	Dominating	1	1

Abbreviations:

AP: antipsychotic medication; FI: family intervention; ICER: incremental cost-effectiveness ratio; NMB: net monetary benefit; WTP: willingness-to-pay.

© 2020 Jin H et al. *JAMA Network Open.*

eAppendix 7. Comparing Results With Published Literature

7.1 Topic A – Interventions for patients at CHR

No existing models were identified for Topic A.[44] Our rapid review identified one recently published clinical trial carried out in the Netherlands which compared the clinical and economic outcomes for people at CHR who received routine care only with people who received routine care plus CBT.[45] The economic findings were similar to the WDM results presented: over a 4-year follow-up period, use of CBT was associated with lower costs (mean difference=-US $5,777 per person at CHR), and a marginal non-significant difference in QALY gains (mean difference=0.12, 95% CI: 0.00-0.25) compared with routine care.

7.2 Topic B – Interventions for people with acute psychosis

No existing models were identified for Topic B.[44] Our rapid review identified one trial-based economic evaluation conducted by McCrone and colleagues in London which shows similar findings to our analysis: use of CRHT results in lower costs compared with hospital admission alone.[37] McCrone *et al.* did not report the impact of receiving alternative services on patients' HRQoL or mortality.

7.3 Topic C – First-line oral antipsychotic for people with FEP

Fifty-three models assessing antipsychotics were identified,[44] of which only one specifically assessed first-line antipsychotics for preventing relapse for people with early schizophrenia – this is the model developed by the NICE schizophrenia GDG.[16] Both the results of the NICE model and the schizophrenia WDM found that no antipsychotic can be considered to be clearly more cost-effective compared with the other options, although the results from both models suggest that olanzapine is one of the most cost-effective antipsychotics. However, the results of the NICE model and the schizophrenia WDM differ for amisulpride. The NICE model suggests amisulpride was the least cost-effective option whilst the schizophrenia WDM found it to be the most cost-effective option. This is likely to be due to differences in the input data used. The clinical effectiveness data used in the NICE model was obtained from a network meta-analysis (NMA) conducted by the NICE schizophrenia GDG.[16] According to the results of this NMA, amisulpride is associated with the second highest probability of relapse (second only to haloperidol). However, the studies included in the NICE NMA were obtained from a systematic review conducted in 2008 and since then, two further NMAs which included additional trials have been published, and both analyses showed amisulpride to be associated with one of the lowest probabilities of all-cause discontinuation and relapse rate.[17,46]

7.4 Topic D – Family intervention for people with FEP

Three models were identified which compared the cost-effectiveness of antipsychotic alone versus antipsychotic plus family intervention and CBT or plus family intervention alone.[47-49] None of these models assessed the option of using family intervention alone. All three models suggested that antipsychotic medication plus family intervention is cost-effective compared with antipsychotic alone, which is consistent with the results of WDM.

7.5 Topic E – First-line oral antipsychotics for people with TRS

Four models assessing antipsychotics for people with TRS were identified.[50-53] Of these four models, one did not include clozapine, therefore its findings are not comparable to our study.[50] The other three models compared clozapine with chlorpromazine and haloperidol, and all of them suggested that clozapine dominated chlorpromazine and haloperidol.[51-53] This is consistent with the findings of the schizophrenia WDM, which indicated that clozapine is the most cost-effective antipsychotic for patients with TRS.

eReferences

1. Fusar-Poli P, Byrne M, Badger S, Valmaggia LR, McGuire PK. Outreach and support in south London (OASIS), 2001-2011: ten years of early diagnosis and treatment for young individuals at high clinical risk for psychosis. *European Psychiatry.* 2013;28(5):315-326.
2. Whale R, Harris M, Kavanagh G, et al. Effectiveness of antipsychotics used in first-episode psychosis: a naturalistic cohort study. *The British Journal of Psychiatry Open.* 2016;2(5):323-329.
3. Revier CJ, Reininghaus U, Dutta R, et al. Ten-Year Outcomes of First-Episode Psychoses in the MRC AESOP-10 Study. *The Journal of nervous and mental disease.* 2015;203(5):379-386.
4. Birchwood M, Connor C, Lester H, et al. Reducing duration of untreated psychosis: care pathways to early intervention in psychosis services. *The British Journal of Psychiatry.* 2013;203(1):58-64.
5. Schlosser DA, Jacobson S, Chen Q, et al. Recovery from an at-risk state: clinical and functional outcomes of putatively prodromal youth who do not develop psychosis. *Schizophrenia Bulletin.* 2012;38(6):1225-1233.
6. Alvarez-Jimenez M, Gleeson JF, Henry LP, et al. Prediction of a single psychotic episode: a 7.5-year, prospective study in first-episode psychosis. *Schizophrenia Research.* 2011;125(2-3):236-246.
7. Faerden A, Nesvag R, Marder SR. Definitions of the term 'recovered' in schizophrenia and other disorders. *Psychopathology.* 2008;41(5):271-278.
8. Office for National Statistics. National life tables, UK: 2015 to 2017. 2018. https://www.ons.gov.uk/releases/nationallifetablesuk2015to2017. Accessed 12th Dec 2019.
9. NHS England. *Implementing the Early Intervention in Psychosis Access and Waiting Time Standard: Guidance.* London, UK: NHS England; 2016.
10. Patel R, Shetty H, Jackson R, et al. Delays before Diagnosis and Initiation of Treatment in Patients Presenting to Mental Health Services with Bipolar Disorder. *PLoS One.* 2015;10(5):1-17.
11. Howes OD, Vergunst F, Gee S, McGuire P, Kapur S, Taylor D. Adherence to treatment guidelines in clinical practice: study of antipsychotic treatment prior to clozapine initiation. *British Journal of Psychiatry.* 2012;201(6):481-485.
12. NHS England. *Report of the early intervention in psychosis audit.* London, UK: NHS England; 2016.
13. Green CEL, McGuire PK, Ashworth M, Valmaggia LR. Outreach and Support in South London (OASIS). Outcomes of non-attenders to a service for people at high risk of psychosis: the case for a more assertive approach to assessment. *Psychological Medicine.* 2010;41(2):243-250.
14. NHS Digital. Prescription Cost Analysis - England 2016. 2017. https://digital.nhs.uk/data-and-information/publications/statistical/prescription-cost-analysis/prescription-cost-analysis-england-2016. Accessed 3rd March 2016.
15. Hutton P, Taylor PJ. Cognitive behavioural therapy for psychosis prevention: a systematic review and meta-analysis. *Psychological Medicine.* 2014;44(3):449-468.
16. National Collaborating Centre for Mental Health. *Psychosis and schizophrenia in adults: prevention and management. NICE guideline (CG178).* London, UK: The British Psychological Society and The Royal College of Psychiatrists; 2014.
17. Zhao YJ, Lin L, Teng M, et al. Long-term antipsychotic treatment in schizophrenia: systematic review and network meta-analysis of randomised controlled trials. *The British Journal of Psychiatry Open.* 2016;2(1):59-66.
18. Legge SE, Hamshere M, Hayes RD, et al. Reasons for discontinuing clozapine: A cohort study of patients commencing treatment. *Schizophrenia Research.* 2016;174(1-3):113-119.
19. Samara MT, Dold M, Gianatsi M, et al. Efficacy, Acceptability, and Tolerability of Antipsychotics in Treatment-Resistant Schizophrenia: A Network Meta-analysis. *JAMA Psychiatry.* 2016;73(3):199-210.

20. Lin L, Zhao YJ, Zhou HJ, et al. Comparative cost-effectiveness of 11 oral antipsychotics for relapse prevention in schizophrenia within Singapore using effectiveness estimates from a network meta-analysis. *International Clinical Psychopharmacology.* 2016;31(2):84-92.
21. Essali A, Al-Haj Haasan N, Li C, Rathbone J. Clozapine versus typical neuroleptic medication for schizophrenia. *Cochrane Database of Systematic Reviews.* 2009;21(1):1-213.
22. Mitchell AJ, Vancampfort D, De Herdt A, Yu W, De Hert M. Is the prevalence of metabolic syndrome and metabolic abnormalities increased in early schizophrenia? A comparative meta-analysis of first episode, untreated and treated patients. *Schizophrenia Bulletin.* 2013;39(2):295-305.
23. van Winkel R, De Hert M, Wampers M, et al. Major changes in glucose metabolism, including new-onset diabetes, within 3 months after initiation of or switch to atypical antipsychotic medication in patients with schizophrenia and schizoaffective disorder. *The Journal of clinical psychiatry.* 2008;69(3):472-479.
24. Davies A, Vardeva K, Loze J-Y, L'Italien GJ, Sennfalt K, van Baardewijk M. Cost-effectiveness of atypical antipsychotics for the management of schizophrenia in the UK. *Current Medical Research and Opinion.* 2008;24(11):3275-3285.
25. Gillies CL, Lambert PC, Abrams KR, et al. Different strategies for screening and prevention of type 2 diabetes in adults: cost effectiveness analysis. *British Medical Journal.* 2008;336(7654):1180-1185.
26. Ara R, Brazier JE. Populating an economic model with health state utility values: moving toward better practice. *Value in Health.* 2010;13(5):509-518.
27. Addington J, Penn D, Woods SW, Addington D, Perkins DO. Social functioning in individuals at clinical high risk for psychosis. *Schizophrenia Research.* 2008;99(1-3):119-124.
28. Lenert LA, Sturley AP, Rapaport MH, Chavez S, Mohr PE, Rupnow M. Public preferences for health states with schizophrenia and a mapping function to estimate utilities from positive and negative symptom scale scores. *Schizophrenia Research.* 2004;71(1):155-165.
29. Clarke P, Gray A, Holman R. Estimating utility values for health states of type 2 diabetic patients using the EQ-5D (UKPDS 62). *Medical Decision Making.* 2002;22(4):340-349.
30. Curtis J, Burns A. *Unit Costs of Health and Social Care 2016.* Kent, UK: Personal Social Services Research Unit, University of Kent, Canterbury; 2016.
31. Akhtar W, Chung Y. Saving the NHS one blood test at a time. *BMJ quality improvement report.* 2014;2(2):1-2.
32. Scarborough P, Bhatnagar P, Wickramasinghe KK, Allender S, Foster C, Rayner M. The economic burden of ill health due to diet, physical inactivity, smoking, alcohol and obesity in the UK: an update to 2006-07 NHS costs. *Journal of Public Health.* 2011;33(4):527-535.
33. Joint Formulary Committee. *British National Formulary.* 69 ed. London, UK: BMJ Group and Pharmaceutical Press; 2016.
34. Alva ML, Gray A, Mihaylova B, Leal J, Holman RR. The impact of diabetes-related complications on healthcare costs: new results from the UKPDS (UKPDS 84). *Diabetic Medicine.* 2015;32(4):459-466.
35. Department of Health. NHS reference costs 2016 to 2017. In: UK, London: Department of Health,; 2017: https://www.gov.uk/government/publications/nhs-reference-costs-2016-to-2017. Accessed 15th May 2018.
36. Department of Health. NHS reference costs 2012 to 2013. In: London, UK: Department of Health; 2013: https://www.gov.uk/government/publications/nhs-reference-costs-2012-to-2013. Accessed 15th May 2018.
37. McCrone P, Johnson S, Nolan F, et al. Economic evaluation of a crisis resolution service: a randomised controlled trial. *Epidemiology and Psychiatric Sciences.* 2009;18(1):54-58.
38. Munro J. Hospital treatment and management in relapse of schizophrenia in the UK: associated costs. *The Psychiatrist.* 2011;35(3):95-100.

© 2020 Jin H et al. *JAMA Network Open.*

39. Office for National Statistics. Deaths registered in England and Wales (Series DR): 2015. 2016. https://www.ons.gov.uk/releases/deathsregisteredinenglandandwalesseriesdr2013. Accessed 16th April 2017.
40. NHS National End of Life Care Programme. *Reviewing end of life care costing information to inform the QIPP End of Life Care Workstream.* London, UK: National End of Life Care Programme; 2012.
41. Fusar-Poli P, Bonoldi I, Yung AR, et al. Predicting psychosis: meta-analysis of transition outcomes in individuals at high clinical risk. *Archives Of General Psychiatry.* 2012;69(3):220-229.
42. Curtis J, Burns A. *Unit Costs of Health and Social Care 2013.* Kent, UK: Personal Social Services Research Unit, University of Kent, Canterbury; 2013.
43. Henderson C, Knapp M, Yeeles K, et al. Cost-Effectiveness of Financial Incentives to Promote Adherence to Depot Antipsychotic Medication: Economic Evaluation of a Cluster-Randomised Controlled Trial. *PLoS One.* 2015;10(10):1-18.
44. Jin H, Tappenden P, Robinson S, et al. A systematic review of economic models across the entire schizophrenia pathway. *PharmacoEconomics.* 2020.
45. Ising HK, Lokkerbol J, Rietdijk J, et al. Four-Year Cost-effectiveness of Cognitive Behavior Therapy for Preventing First-episode Psychosis: The Dutch Early Detection Intervention Evaluation (EDIE-NL) Trial. *Schizophrenia Bulletin.* 2017;43(2):365-374.
46. Leucht S, Cipriani A, Spineli L, et al. Comparative efficacy and tolerability of 15 antipsychotic drugs in schizophrenia: a multiple-treatments meta-analysis. *The Lancet.* 2013;382(9896):951-962.
47. Gutierrez-Recacha P, Chisholm D, Haro JM, Salvador-Carulla L, Ayuso-Mateos JL. Cost-effectiveness of different clinical interventions for reducing the burden of schizophrenia in Spain. *Acta Psychiatrica Scandinavica.* 2006;432:29-38.
48. Phanthunane P. Cost-effectiveness of pharmacological and psychosocial interventions for schizophrenia. *Cost Effectiveness and Resource Allocation.* 2011;9(6):1-9.
49. Anh NQ, Linh BN, Ha NT, Phanthunane P, Huong NT. Schizophrenia interventions in Vietnam: Primary results from a cost-effectiveness study. *Global Public Health.* 2015;10:S21-S39.
50. Kim B-R-M, Lee T-J, Lee H-J, Park B-H, Yang B-M. Cost-Effectiveness of Sertindole among Atypical Antipsychotics in the Treatment of Schizophrenia in South Korea. *Value in Health Regional Issues.* 2012;1(1):59-65.
51. Davies LM, Drummond MF. Assessment of costs and benefits of drug therapy for treatment-resistant schizophrenia in the United Kingdom. *British Journal of Psychiatry.* 1993;162:38-42.
52. Glennie J. *Pharmacoeconomic evaluations of clozapine in treatment-resistant schizophrenia and risperidone in chronic schizophrenia.* Ottawa, Canada: Canadian Coordinating Office for Health Technology Assessment; 1997.
53. Oh PI, Iskedjian M, Addis A, Lanctot K, Einarson TR. Pharmacoeconomic evaluation of clozapine in treatment-resistant schizophrenia: a cost-utility analysis. *Canadian Journal of Clinical Pharmacology.* 2001;8(4):199-206.

© 2020 Jin H et al. *JAMA Network Open.*
